# Effects of socioeconomic status on esophageal adenocarcinoma stage at diagnosis, receipt of treatment, and survival: A population-based cohort study

**DOI:** 10.1371/journal.pone.0186350

**Published:** 2017-10-11

**Authors:** Hla-Hla Thein, Kika Anyiwe, Nathaniel Jembere, Brian Yu, Prithwish De, Craig C. Earle

**Affiliations:** 1 Dalla Lana School of Public Health, University of Toronto, Toronto, Ontario, Canada; 2 Institute for Clinical Evaluative Sciences, Toronto, Ontario, Canada; 3 Western University, Medical Science, London, Ontario, Canada; 4 Cancer Care Ontario, Toronto, Ontario, Canada; 5 Ontario Institute for Cancer Research, Toronto, Ontario, Canada; University Hospital Llandough, UNITED KINGDOM

## Abstract

The incidence of esophageal adenocarcinoma (EAC) is increasing worldwide and has overtaken squamous histology in occurrence. We studied the impact of socioeconomic status (SES) on EAC stage at diagnosis, receipt of treatment, and survival. A population-based retrospective cohort study was conducted using Ontario Cancer Registry-linked administrative health data. Multinomial logistic regression was used to examine the association between SES (income quintile) and stage at EAC diagnosis and EAC treatment. Survival times following EAC diagnosis were estimated using Kaplan-Meier method. Cox proportional-hazards regression analysis was used to examine the association between SES and EAC survival. Between 2003–2012, 2,125 EAC cases were diagnosed. Median survival for the lowest-SES group was 10.9 months compared to 11.6 months for the highest-SES group; the 5-year survival was 9.8% vs. 15.0%. Compared to individuals in the highest-SES group, individuals in the lowest-SES category experienced no significant difference in EAC treatment (91.6% vs. 93.3%, *P* = 0.314) and deaths (78.9% vs. 75.6%, *P* = 0.727). After controlling for covariates, no significant associations were found between SES and cancer stage at diagnosis and EAC treatment. Additionally, after controlling for age, gender, urban/rural residence, birth country, health region, aggregated diagnosis groups, cancer stage, treatment, and year of diagnosis, no significant association was found between SES and EAC survival. Moreover, increased mortality risk was observed among those with older age (*P* = 0.001), advanced-stage of EAC at diagnosis (*P* < 0.001), and those receiving chemotherapy alone, radiotherapy alone, or surgery plus chemotherapy (*P* < 0.001). Adjusted proportional-hazards model findings suggest that there is no association between SES and EAC survival. While the unadjusted model suggests reduced survival among individuals in lower income quintiles, this is no longer significant after adjusting for any covariate. Additionally, there is an apparent association between SES and survival when considering only those individuals diagnosed with stage 0-III EAC. These analyses suggest that the observed direct relationship between SES and survival is explained by patient-level factors including receipt of treatment, something that is potentially modifiable.

## Introduction

Esophageal adenocarcinoma (EAC) is predominantly a disease of the distal esophagus and gastroesophageal junction. EAC incidence has greatly increased over the past three decades, gaining global relevance as a clinically important cancer [1–4]. In Ontario, new cases of EAC per 100,000 persons have nearly tripled from 0.79 in 1982 to 2.26 in 2008 representing a 4% per year increase in EAC incidence [5]. Barrett’s esophagus is the presumed precursor lesion of EAC, progressing to EAC in a small percentage of patients [6]. Epidemiological studies have identified additional important risk factors for the development of EAC, including age, gender, race, duration of gastroesophageal reflux disease (GERD) symptoms, smoking, and obesity (elevated body mass index) [6–14].

EAC is a rare but highly fatal cancer, accounting for 1% of all cancers diagnosed in Ontario in 2009. Despite improvements in the care of patients with EAC, overall mortality remains high, with a 5-year relative survival of 14% between 2006 and 2008 [15]. Poor mortality is thought to occur because most patients with EAC present with advanced-stage disease, after symptoms of dysphagia are already present, and are not eligible for highly effective and usually curative endoscopic therapies [6]. Socioeconomic status (SES) may also affect how individuals with Barrett's esophagus or EAC follow cancer screening and treatment recommendations. People with higher levels of income and education are more likely to participate in cancer screening and treatment. Lower SES was found to be associated with cancer stage at diagnosis, longer health care delay, and varying receipt of treatment for esophageal cancer [16].

Studies have shown that compared to those with high SES, cancer patients with low SES have an increased risk of mortality, even within the context of universal health care [17, 18]. Studies regarding the relationship between SES and survival have had conflicting results. Previous studies in Canada (head and neck cancer) and in Ontario (hepatocellular carcinoma) have demonstrated that lower SES is associated with worse survival outcomes [19, 20]. The relationship between SES and survival has yet to be explored for EAC in Canada.

Despite advances in cancer treatment, improvements in survival outcomes have not been equally distributed among all socioeconomic groups. Multiple theories have been proposed for the observed survival advantage experienced by people at higher SES levels. It has been proposed that higher SES is associated with seeking treatment earlier in disease progression, whereas lower SES is associated with delayed treatment seeking until the cancer has become symptomatic and incurable [16, 21]. Other theories suggest that those at higher SES have better access to treatment and care [19, 22]. and experience lower levels of comorbidity, leading to reduced overall as well as cause-specific cancer mortality [23].

Regional variation of EAC incidence is also important in order to provide further care to areas with greater health burdens. It is important to study the effect of SES on EAC survival and regional variation in order to further stratify the at-risk population and optimize the current EAC screening opportunities in Ontario. The purpose of this study is to assess the impact of SES on cancer stage at diagnosis, receipt of treatment, regional variation, and survival among a cohort of cases with EAC.

## Materials and methods

### Study design and population

This population-based retrospective cohort study considered all eligible patients 18 years of age and older who were diagnosed with EAC in Ontario between 1 January 1993 and 31 December 2012. We identified individuals in the Ontario Cancer Registry (OCR) with an ICD-9 code 150 and histology ICD-O-3 codes 8140–8575 (S1 Table). Individuals whose date of EAC diagnosis was the same as the date of death or individuals whose EAC was not the primary site were excluded.

### Data sources

For this study, we used the following databases: the OCR, the Ontario Health Insurance Plan (OHIP), the Registered Persons Database, and the Canadian Institute for Health Information Discharge Abstract Database (DAD) and the National Ambulatory Care Reporting System (NACRS). The OCR contains all cancer incidence (new cases) and mortality (deaths) in Ontario from 1964 onward. OHIP data contains the records of all physician billings for inpatient and outpatient visits and procedures starting from January 1991. The Registered Persons Database contains demographic and address information for all people registered for provincial government-sponsored health insurance coverage. The DAD contains demographic, clinical, and administrative data for hospital admissions and day surgeries in Ontario starting from 1991, and the NACRS contains administrative, demographic, clinical, and financial data for hospital-based ambulatory care. To track treatments use, we searched for claims for surgical resection, chemotherapy and radiotherapy for EAC from OHIP, DAD and NACRS fee codes. We also searched for fee codes from OHIP, DAD and NACRS for the following palliative procedures: esophageal dilation, drainage, esophageal stenting, laser debulking of tumor, and palliative care. See Supporting Information S2 Table.

Data were provided by the Institute for Clinical Evaluative Sciences which contain the health records for the roughly 14 million residents of Ontario. All data provided on EAC cases were post cancer diagnosis. No health records prior to cancer diagnosis were provided. We therefore could not assess screening for EAC (endoscopy and biopsy), prior Barrett’s esophagus diagnosis, or prior cancer diagnosis.

### Study variables

Variables considered in the analyses included SES (income quintile, Q1-Q5), age group (<50, 50–54, 55–59, 60–64, 65–69, 70–74, 75–79, 80–84, ≥85 years), gender (male, female), residence (rural, urban), birth country (outside of Canada, Canada), Ontario health region (Erie St. Clair, South West, Waterloo Wellington, Hamilton Niagara Haldimand Brant, Central West, Mississauga, Toronto Central, Central, Central East, South East, Champlain, North Simcoe, North East, North West), aggregated diagnosis groups (ADGs), stage at EAC diagnosis (Stage 0 [earliest stage of EAC, also called high-grade dysplasia; cancer cells are found only in the epithelium], Stage I, Stage II, Stage III, Stage IV), treatment for EAC (categorized exclusively as surgery, chemotherapy or radiotherapy alone, surgery plus chemotherapy, surgery plus radiotherapy, chemotherapy plus radiotherapy, surgery plus chemotherapy plus radiotherapy, and no treatment), year of EAC diagnosis (1993–1997, 1998–2002, 2003–2007, 2008–2012), and date of death. Classification of malignant tumors based on the American Joint Committee on Cancer TNM staging [extent of the tumor (T), extent of spread to the lymph nodes (N), and presence of metastasis (M)] [24] was used in the OCR from 2003 onwards.

Individual-level SES was not available therefore area-level SES was used as a surrogate. Area-level SES was quantified using median neighbourhood household income. Median neighbourhood household income was determined through linking of postal codes to Canadian census data; income was categorized into quintiles corresponding to income status of neighbourhoods. Income quintile 1 represents the lowest 20% of neighborhoods and income quintile 5 represents the most well-off 20% of neighbourhoods.

Ontario is currently divided into 14 health regions which plan and fund local health care. Each Local Health Integration Network’s mandate is to make the health system more efficient and improve access to quality care [25]. Patients’ local health regions were used as a factor to explain regional health care service and availability.

We classified patient comorbidity using the Johns Hopkins Adjusted Clinical Groups (ACG) case-mix system, which has been validated in the United States [26, 27] and in Canada [28, 29]. The ACG system measures individuals’ morbidity by grouping individuals based on their age and gender and all medical diagnoses over a given time period. For this study, we used Ontario inpatient Discharge Abstract Database and outpatient OHIP diagnosis codes from the year prior to the date of EAC diagnosis to estimate case-mix. Patients grouped into 32 different ADG categories method may be useful for comorbidity adjustment in administrative health care data when comparing morbidity, mortality, or health care utilization and costs [30–32].

### Outcome measure

The primary outcome for our study was survival time after EAC diagnosis. Survival time was calculated using the time between death and date of diagnosis. If no death was observed during the follow up period, the patient was censored. As secondary outcomes, stage of diagnosis, receipt of EAC treatment and health region were compared by SES.

### Statistical analysis

Overall EAC patient characteristics and patient characteristics by SES (income quintile) and by year of EAC diagnosis from 1993 to 2012 were tabulated. Chi-squared tests were used to examine the association between income quintile and relevant variables as mentioned above. The following survival analyses were determined for study periods 2003–2012 due to the availability of EAC stage. Median survival times (months, with interquartile range [IQR]), and 1-year, 3-year, and 5-year survival (95% confidence intervals [CIs]) after EAC diagnosis overall, and stratified by income quintile and other covariates were estimated using Kaplan–Meier survival analysis. Differences between survival times were assessed using log-rank tests.

Multinomial logistic regression analysis was used to examine the association between income quintile and stage at EAC diagnosis, yielding odds ratios and corresponding 95% CIs of stage II, stage III, or stage IV relative to stage 0-I EAC at diagnosis, using income quintile 5 as the reference. Multinomial logistic regression analysis was also used to assess the association between income quintile and receiving treatment for EAC relative to no treatment as well as between income quintile and patients’ health regions relative to Central region. We adjusted for potential confounding covariates, including age, gender, urban/rural residence, birth country, health region (to assess the association between income quintile and cancer stage and treatment), ADGs, and year of EAC diagnosis. Cancer stage at diagnosis was included as a covariate to assess the association between income quintile and EAC treatment and health region.

Cox proportional-hazards regression analysis using unadjusted (univariate) and adjusted (multivariate) models were used to assess the association between income quintile and EAC survival. The main exposure variable we assessed was SES. Age and gender were evaluated as confounders of SES, along with urban/rural residence, birth country, health region, ADGs, cancer stage at diagnosis, treatment, and year of EAC diagnosis. EAC treatment was modeled as a time-dependent variable within the proportional-hazards regression model; treatment status changed from 0 to 1 based on the treatment date (if cases received treatment during illness). The results are reported as hazard ratios (HRs) with 95% CIs. Additionally, the association between income quintile and survival was assessed, considering only those individuals diagnosed with stage 0-III EAC (excluding advanced-stage IV).

A two-sided *P*-value of < 0.05 was considered to be statistically significant. Statistical analyses were conducted using SAS version 9.4 (SAS Institute Inc., Cary, NC, USA) and STATA version 12.0 (Stata Corporation, College Station, TX) statistical software applications.

### Sensitivity analysis

To use all available EAC cases (1993–2012) for the association between income quintile and outcomes, multiple imputation was used to impute values for variables with a significant portion of missing data. Variables which were imputed were income quintile, urban/rural residence, birth country, and cancer stage at EAC diagnosis. Five independent draws from an imputation model were used to create five completed data sets and results were combined to obtain one imputation inference [33]. Multiple Imputation procedure by logistic regression was used in a sequential process to generate monotone patterns (PROC MI with LOGISTIC in the MONOTONE statement) [33–35].

### Ethics approval

Ethics approval for the study was granted by the University of Toronto Health Sciences Research Ethics Board. Informed consent was not obtained because this secondary analysis accessed existing de-identified data; consent was therefore deemed to be neither feasible nor necessary.

## Results

### Baseline characteristics of patients diagnosed with EAC

A flow chart of the study population can be found in S1 Fig. Overall sociodemographic and clinical characteristics of patients diagnosed with EAC and by year of EAC diagnosis are summarized in S3 Table. In Ontario during the period 1993–2012, 5,382 cases were diagnosed principally as EAC. Overall, there was an increase in EAC diagnosis from 16.0% during the period 1993–1997 to 35.1% during 2008–2012. Overall, the 5,382 patients were evenly distributed across income quintile categories (i.e. 20.3%, 20.6%, 20.1%, 19.6%, and 19.0% from income quintile 1 to 5).

The proportion of cases diagnosed among patients aged 60–64 years increased from 11.8% during 1993–1997 to 16.2% during 2008–2012; conversely, the proportion of cases diagnosed among those aged 70–74 years decreased (from 18.0% to 13.2%) during this same time period. The majority of patients were male (84%), with a male to female ratio of about 5:1. The highest number of EAC cases occurred in persons with ADGs 11 and above (which increased from 43.2% during 1993–1997 to 49.1% during 2008–2012) (S3 Table).

Stage at EAC diagnosis was available from 2003 to 2012; 145 (2.7%) people were diagnosed with stage 0-I, while 445 (8.3%) were stage II, 515 (9.6%) were stage III, 1,020 (19.0%) were stage IV, and 3,257 (60.5%) were unknown stage. The percentage of people who received treatment increased throughout the study period, for all therapies except for surgical treatment. The proportion of patients receiving surgical treatment decreased from 28.6% in 1993–1997 to 9.2% in 2008–2012. Deaths among patients with EAC decreased from 97% (n = 833) during 1993–1997 to 68.4% (n = 1,292) during 2008–2012 (S3 Table).

### Population with EAC diagnosis by SES

Descriptive characteristics of the 5,382 individuals with EAC stratified by income quintile are summarized in Table 1. Urban/rural residence (*P* < 0.001), Ontario health region (*P* < 0.001), and receiving EAC treatment (*P* = 0.048) were the factors that were significant when stratified by income quintiles. People within lower income quintiles were less likely to receive treatment (no treatment: 41.5% and 39.4% for lower income quintiles 1 and 2, respectively) than those within higher income quintiles (36.3% to 34.1% for income quintiles 3–5, *P* = 0.002). In addition, people in lower to mid SES groups (84.1% to 85.8%) were more likely to die than those in higher income quintiles (81.7% to 82.3%, *P* = 0.066), although this was not statistically significant.

**Table 1:** Association of socioeconomic status with potential covariates among population with esophageal adenocarcinoma, 1993–2012.

Variable	Income Quintile 1	Income Quintile 2	Income Quintile 3	Income Quintile 4	Income Quintile 5	Missing	*P*-value
	N (%)	N (%)	N (%)	N (%)	N (%)	N (%)	
Total N (%)	1092 (20.3)	1108 (20.6)	1084 (20.1)	1054 (19.6)	1024 (19.0)	20 (0.4)	
Age group (years)							
<50	87 (8.0)	80 (7.2)	105 (9.7)	101 (9.6)	80 (7.8)	‒	
50–54	91 (8.3)	92 (8.3)	91 (8.4)	95 (9.0)	89 (8.7)	0	
55–59	121 (11.1)	120 (10.8)	105 (9.7)	117 (11.1)	119 (11.6)	‒	
60–64	162 (14.8)	158 (14.3)	128 (11.8)	140 (13.3)	151 (14.8)	‒	
65–69	161 (14.7)	162 (14.6)	168 (15.5)	161 (15.3)	150 (14.7)	‒	
70–74	169 (15.5)	175 (15.8)	159 (14.7)	146 (13.9)	140 (13.7)	‒	
75–79	129 (11.8)	136 (12.3)	153 (14.1)	136 (12.9)	136 (13.3)	0	
80–84	98 (9.0)	106 (9.6)	109 (10.1)	100 (9.5)	93 (9.1)	‒	
≥85	74 (6.8)	79 (7.1)	66 (6.1)	58 (5.5)	66 (6.5)	‒	0.780
Sex							
Male	907 (83.1)	906 (81.8)	910 (84.0)	899 (85.3)	881 (86.0)	17 (85.0)	
Female	185 (16.9)	202 (18.2)	174 (16.1)	155 (14.7)	143 (14.0)	‒	0.098
Residence							
Rural	224 (20.5)	204 (18.4)	192 (17.7)	207 (19.6)	191 (18.7)	8 (40.0)	
Urban	868 (79.5)	904 (81.6)	892 (82.3)	847 (80.4)	833 (81.4)	9 (45.0)	
Missing	0	0	0	0	0	‒	**<0.001**
Birth country							
Outside of Canada	229 (21.0)	213 (19.2)	221 (20.4)	204 (19.4)	215 (21.0)	‒	
Canada	694 (63.6)	697 (62.9)	677 (62.5)	639 (60.6)	616 (60.2)	14 (70.0)	
Missing	169 (15.5)	198 (17.9)	186 (17.2)	211 (20.0)	193 (18.9)	‒	0.381
Ontario Health Region							
Erie St. Clair	62 (5.7)	56 (5.1)	55 (5.1)	54 (5.1)	58 (5.7)	0	
South West	96 (8.8)	117 (10.6)	116 (10.7)	96 (9.1)	76 (7.4)	‒	
Waterloo Wellington	53 (4.9)	80 (7.2)	54 (5.0)	57 (5.4)	67 (6.5)	0	
Hamilton Niagara Haldimand Brant	163 (14.9)	156 (14.1)	152 (14.0)	161 (15.3)	132 (12.9)	‒	
Central West	19 (1.7)	45 (4.1)	44 (4.1)	47 (4.5)	28 (2.7)	0	
Mississauga	26 (2.4)	34 (3.1)	51 (4.7)	61 (5.8)	66 (6.5)	‒	
Toronto Central	91 (8.3)	52 (4.7)	55 (5.1)	47 (4.5)	97 (9.5)	‒	
Central	54 (5.0)	72 (6.5)	62 (5.7)	92 (8.7)	103 (10.1)	‒	
Central East	134 (12.3)	128 (11.6)	128 (11.8)	116 (11.0)	84 (8.2)	‒	
South East	113 (10.4)	86 (7.8)	84 (7.8)	79 (7.5)	57 (5.6)	‒	
Champlain	90 (8.2)	120 (10.8)	134 (12.4)	107 (10.2)	130 (12.7)	‒	
North Simcoe	52 (4.8)	45 (4.1)	59 (5.4)	50 (4.7)	50 (4.9)	‒	
North East	117 (10.7)	93 (8.4)	58 (5.4)	55 (5.2)	44 (4.3)	‒	
North West	22 (2.0)	24 (2.2)	32 (3.0)	32 (3.0)	32 (3.1)	0	**<0.001**
ADG							
0	10 (0.9)	6 (0.5)	7 (0.7)	6 (0.6)	6 (0.6)	‒	
1–3	52 (4.8)	47 (4.2)	37 (3.4)	68 (6.5)	48 (4.7)	‒	
4–7	197 (18.0)	223 (20.1)	226 (20.9)	195 (18.5)	221 (21.6)	‒	
8–10	316 (28.9)	298 (26.9)	280 (25.8)	271 (25.7)	251 (24.5)	‒	
11+	517 (47.3)	534 (48.2)	534 (49.3)	514 (48.8)	498 (48.6)	9 (45.0)	0.091
Stage at EAC diagnosis							
Stage 0-I	26 (2.4)	26 (2.4)	29 (2.7)	35 (3.3)	29 (2.8)	0	
Stage II	88 (8.1)	92 (8.3)	95 (8.8)	91 (8.6)	77 (7.5)	‒	
Stage III	98 (9.0)	93 (8.4)	96 (8.9)	110 (10.4)	115 (11.2)	‒	
Stage IV	205 (18.8)	225 (20.3)	204 (18.8)	198 (18.8)	184 (18.0)	‒	
Unknown	675 (61.8)	672 (60.7)	660 (60.9)	620 (58.8)	619 (60.5)	11 (55.0)	0.834
EAC treatment							
Surgery alone	161 (14.7)	157 (14.2)	173 (16.0)	170 (16.1)	153 (14.9)	‒	
Chemotherapy alone	91 (8.3)	117 (10.6)	104 (9.6)	100 (9.5)	109 (10.6)	‒	
Radiotherapy alone	90 (8.2)	92 (8.3)	86 (7.9)	77 (7.3)	102 (10.0)	3 (15.0)	
Surgery + chemotherapy	73 (6.7)	64 (5.8)	77 (7.1)	83 (7.9)	82 (8.0)	‒	
Surgery + radiotherapy	9 (0.8)	10 (0.9)	11 (1.0)	14 (1.3)	3 (0.3)	‒	
Chemotherapy + radiotherapy	125 (11.5)	127 (11.5)	130 (12.0)	120 (11.4)	110 (10.7)	‒	
Surgery + chemotherapy + radiotherapy	90 (8.2)	104 (9.4)	110 (10.2)	116 (11.0)	116 (11.3)	‒	
No treatment	453 (41.5)	437 (39.4)	393 (36.3)	374 (35.5)	349 (34.1)	11 (55.0)	**0.048**
Palliative care	698 (63.9)	702 (63.4)	686 (63.3)	653 (62.0)	645 (63.0)	14 (70.0)	0.928
Year of EAC diagnosis							
1993–1997	183 (16.8)	159 (14.4)	182 (16.8)	163 (15.5)	166 (16.2)	6 (30.0)	
1998–2002	237 (21.7)	244 (22.0)	238 (22)	204 (19.4)	225 (22.0)	‒	
2003–2007	288 (26.4)	297 (26.8)	314 (29.0)	296 (28.1)	282 (27.5)	7 (35.0)	
2008–2012	384 (35.2)	408 (36.8)	350 (32.3)	391 (37.1)	351 (34.3)	6 (30.0)	0.290
Deaths	937 (85.8)	932 (84.1)	925 (85.3)	861 (81.7)	843 (82.3)	17 (85.0)	0.066

Total N = 5,382. “‒“, counts less than 6 are suppressed. ADG, Aggregated Diagnosis Group; EAC, esophageal adenocarcinoma.

### Survival after EAC diagnosis

Table 2 shows the median, 1-year, 3-year, and 5-year survival estimates. The overall median survival of the population was 11.1 months (IQR: 4.9–28.0). The median survival estimates for income quintiles 1–5 were 10.9 (IQR: 4.3–25.1), 10.9 (IQR: 4.9–22.1), 10.9 (IQR: 4.9–30.4), 11.9 (5.3–33.3), and 11.6 (IQR: 4.7–32.0) months, respectively. Relative increases in median survival were found for patients: who were below 70 years of age compared to those 80 years or above (12.4 to 13.2 months vs. 7.1 to 8.8 months), whose stage at EAC diagnosis was 0-I compared to stage IV (41,2 vs 6.0 months), and who received surgery plus chemotherapy (35.7 months), surgery alone (34.0 months) or surgery plus chemotherapy plus radiotherapy (28.2 months) vs. no treatment (1.6 months).

**Table 2:** Unadjusted survival of people diagnosed with esophageal adenocarcinoma, 2003–2012.

Characteristics	Cases	Events	Survival (Months)	1-Year Survival	3-Year Survival	5-Year Survival
	N (%)	N (%)	Median (IQR)	(%) (95% CI)	(%) (95% CI)	(%) (95% CI)
Overall	2125 (100)	1642 (100)	11.1 (4.9–28.0)	47.5 (45.3–49.6)	20.7 (18.8–22.7)	13.7 (11.9–15.7)
Income quintile						
1 (lowest)	417 (19.7)	329 (20.1)	10.9 (4.3–25.1)	47.0 (42.1–51.8)	16.6 (12.7–20.9)	9.8 (6.3–14.3)
2	436 (20.6)	342 (20.9)	10.9 (4.9–22.1)	45.5 (40.6–50.2)	17.1 (13.3–21.2)	11.0 (7.4–15.2)
3	424 (20.0)	330 (20.2)	10.9 (4.9–30.4)	46.8 (41.9–51.5)	21.5 (17.3–25.9)	15.6 (11.8–20.0)
4	434 (20.5)	329 (20.1)	11.9 (5.3–33.3)	49.5 (44.7–54.2)	24.4 (20.2–28.9)	16.5 (12.4–21.0)
5 (highest)	405 (19.1)	306 (18.7)	11.6 (4.7–32.0)	48.7 (43.7–53.5)	23.5 (19.2–28.2)	15.0 (10.7–20.0)
Age group (years)						
<50	189 (8.9)	144 (8.8)	12.7 (6.2–29.4)	51.3 (43.8–58.3)	22.4 (16.2–29.3)	14.7 (9.2–21.4)
50–54	221 (10.4)	162 (9.9)	12.9 (5.7–30.2)	52.3 (45.4–58.7)	23.5 (17.6–29.8)	16.5 (10.1–24.2)
55–59	262 (12.3)	192 (11.7)	13.2 (5.7–38.2)	52.2 (45.9–58.1)	26.0 (20.4–32.0)	16.1 (10.8–22.4)
60–64	348 (16.4)	247 (15.0)	12.4 (5.5–41.4)	50.4 (45.0–55.6)	27.0 (22.1–32.2)	22.0 (17.0–27.4)
65–69	309 (14.5)	235 (14.3)	12.9 (5.4–28.2)	52.1 (46.3–57.6)	20.7 (15.8–26.0)	11.9 (7.4–17.5)
70–74	269 (12.7)	218 (13.3)	10.1 (3.8–26.2)	43.6 (37.5–49.5)	18.4 (13.6–23.8)	10.8 (6.8–16.0)
75–79	249 (11.7)	206 (12.6)	10.5 (4.2–24.9)	44.9 (38.6–51.0)	17.7 (12.9–23.1)	11.5 (7.1–16.9)
80–84	186 (8.8)	161 (9.8)	7.1 (3.2–16.4)	32.7 (26.0–39.6)	8.5 (4.6–14.0)	3.2 (0.5–11.1)
≥85	92 (4.3)	77 (4.7)	8.8 (4.7–16.2)	36.7 (26.6–46.8)	11.8 (5.7–20.2)	4.4 (0.5–15.6)
Sex						
Male	1828 (86.0)	1402 (85.4)	11.4 (4.9–28.6)	48.3 (45.9–50.6)	21.2 (19.1–23.3)	13.9 (11.9–16.0)
Female	297 (14.0)	240 (14.6)	9.9 (4.4–22.9)	42.8 (37.0–48.4)	18.1 (13.6–23.1)	12.9 (8.6–18.1)
Residence						
Rural	401 (18.9)	301 (18.3)	10.9 (4.9–26.3)	47.3 (42.2–52.2)	19.7 (15.4–24.3)	15.0 (10.8–19.9)
Urban	1724 (81.1)	1341 (81.7)	11.1 (4.8–28.2)	47.5 (45.1–49.9)	21.0 (18.9–23.1)	13.5 (11.5–15.6)
Birth country						
Outside of Canada	340 (21.5)	340 (21.6)	7.1 (3.6–15.3)	33.8 (28.8–38.9)	5.0 (3.0–7.7)	0.6 (0.1–2.0)
Canada	1239 (78.5)	1232 (78.4)	8.0 (3.8–14.3)	33.2 (30.6–35.8)	5.0 (3.9–6.3)	1.1 (0.6–1.8)
Ontario Health Region						
Erie St. Clair	99 (4.7)	76 (4.6)	12.3 (5.4–28.6)	50.4 (40.1–59.9)	22.5 (14.2–32.1)	15.1 (7.5–25.1)
South West	171 (8.1)	148 (9.0)	8.1 (4.4–17.2)	35.6 (28.4–42.8)	12.5 (7.7–18.5)	7.6 (3.5–13.7)
Waterloo Wellington	136 (6.4)	104 (6.3)	10.0 (5.6–26.2)	45.4 (36.8–53.6)	18.0 (11.2–26.1)	12.3 (6.1–20.9)
Hamilton Niagara Haldimand Brant	371 (17.5)	306 (18.6)	9.1 (3.9–20.9)	41.4 (36.3–46.4)	13.7 (10.0–18.0)	7.8 (4.7–11.8)
Central West	47 (2.2)	32 (2.0)	15.0 (6.9–44.3)	55.0 (39.7–67.9)	32.1 (18.7–46.4)	24.1 (11.5–39.2)
Mississauga	55 (2.6)	48 (2.9)	8.3 (3.3–18.3)	32.7 (20.8–45.1)	10.6 (4.0–20.9)	10.6 (4.0–20.9)
Toronto Central	118 (5.6)	86 (5.2)	13.2 (7.2–32.7)	52.2 (42.6–61.0)	23.6 (15.5–32.7)	16.1 (8.6–25.6)
Central	141 (6.6)	97 (5.9)	16.5 (6.3–49.4)	63.3 (54.7–70.7)	30.7 (22.8–39.0)	24.1 (16.4–32.7)
Central East	218 (10.3)	163 (9.9)	12.0 (5.8–34.0)	49.6 (42.7–56.1)	23.7 (17.8–30.0)	16.0 (10.3–23.0)
South East	183 (8.6)	137 (8.3)	11.6 (4.2–30.1)	49.4 (41.8–56.4)	21.7 (15.4–28.8)	15.9 (9.9–23.2)
Champlain	260 (12.2)	200 (12.2)	12.2 (4.0–31.5)	50.7 (44.4–56.7)	23.4 (18.1–29.1)	16.4 (11.2–22.3)
North Simcoe	110 (5.2)	76 (4.6)	13.0 (6.1–47.2)	52.9 (43.1–61.8)	30.1 (20.8–39.8)	21.5 (12.5–32.0)
North East	148 (7.0)	114 (6.9)	10.5 (5.5–26.9)	48.4 (40.0–56.3)	20.0 (13.2–27.8)	8.3 (3.1–16.7)
North West	68 (3.2)	55 (3.4)	9.5 (4.9–24.2)	41.7 (29.7–53.1)	16.7 (8.3–27.6)	10.0 (3.2–21.5)
ADG						
0	14 (0.7)	12 (0.7)	6.1 (3.5–19.7)	28.6 (8.8–52.4)	14.3 (2.3–36.6)	14.3 (2.3–36.6)
1–3	89 (4.2)	67 (4.1)	11.0 (4.9–22.7)	49.2 (38.3–59.2)	18.0 (10.0–28.0)	14.4 (6.5–25.4)
4–7	410 (19.3)	318 (19.4)	11.6 (4.6–25.5)	48.7 (43.7–53.5)	21.1 (16.9–25.6)	10.7 (7.1–15.1)
8–10	587 (27.6)	455 (27.7)	11.0 (5.1–26.1)	47.8 (43.6–51.8)	18.3 (14.9–22.0)	13.2 (9.8–17.2)
11+	1025 (48.2)	790 (48.1)	11.0 (4.9–30.0)	47.0 (43.9–50.1)	22.1 (19.4–25.0)	15.0 (12.3–17.8)
Stage at EAC diagnosis						
Stage 0-I	145 (6.8)	60 (3.7)	41.2 (16.4-NA)	84.4 (77.1–89.6)	57.6 (47.6–66.3)	36.1 (24.2–48.2)
Stage II	445 (20.9)	280 (17.1)	21.7 (10.4–68.9)	70.3 (65.7–74.4)	38.2 (33.3–43.1)	28.2 (23.2–33.5)
Stage III	515 (24.2)	370 22.5)	15.7 (8.5–35.7)	62.4 (58.0–66.5)	24.3 (20.2–28.7)	14.8 (10.9–19.2)
Stage IV	1020 (48.0)	932 (56.8)	6.0 (2.9–12.0)	24.9 (22.3–27.6)	6.2 (4.7–8.0)	3.6 (2.4–5.3)
EAC treatment						
Surgery alone	132 (6.2)	67 (4.1)	34.0 (8.3–91.3)	66.1 (56.8–73.8)	49.2 (39.1–58.6)	37.2 (26.4–48.0)
Chemotherapy alone	112 (5.3)	97 (5.9)	6.0 (3–11.9)	24.3 (16.8–32.7)	9.2 (4.1–16.8)	-
Radiotherapy alone	424 (20.0)	395 (24.1)	5.0 (2.7–9.7)	19.0 (15.3–23.0)	4.6 (2.7–7.2)	2.4 (0.9–5.0)
Surgery + chemotherapy	89 (4.2)	46 (2.8)	35.7 (10.7-NA)	72.1 (61.4–80.4)	49.7 (38.2–60.2)	38.6 (26.4–50.8)
Surgery + radiotherapy	48 (2.3)	42 (2.6)	15.0 (9.5–29.4)	64.6 (49.4–76.3)	20.2 (9.9–33.0)	4.3 (0.4–16.4)
Chemotherapy + radiotherapy	614 (28.9)	538 (32.8)	9.3 (5.6–16.2)	39.4 (35.5–43.3)	8.4 (6.0–11.2)	3.3 (1.6–5.9)
Surgery + chemotherapy + radiotherapy	561 (26.4)	326 (19.9)	28.2 (14.3–70.3)	82.5 (79.1–85.4)	41.7 (37.2–46.2)	28.8 (24.0–33.8)
No treatment	145 (6.8)	131 (8.0)	1.6 (0.7–3.4)	8.2 (4.3–13.9)	4.2 (1.5–9.1)	2.8 (0.7–7.8)
Palliative care	1859 (87.5)	1462 (89.0)	11.5 (5.2–27.5)	48.5 (46.1–50.7)	19.8 (17.9–21.9)	12.3 (10.4–14.3)
Year of EAC diagnosis						
2003–2004	208 (9.8)	184 (11.2)	11.1 (4.2–28.6)	46.3 (39.3–52.9)	19.9 (14.7–25.6)	13.8 (9.5–19.0)
2005–2006	417 (19.6)	371 (22.6)	9.9 (4.6–23.5)	43.4 (38.6–48.2)	17.0 (13.5–20.8)	10.8 (8.0–14.1)
2007–2008	465 (21.9)	404 (24.6)	10.3 (4.5–25.1)	45.1 (40.5–49.6)	18.7 (15.3–22.4)	11.4 (8.6–14.7)
2009–2010	591 (27.8)	459 (28.0)	11.1 (4.9–25.1)	48.4 (44.3–52.4)	19.3 (16.0–22.8)	17.6 (14.2–21.2)
2011–2012	444 (20.9)	224 (13.6)	14.5 (5.6-NA)	53.7 (48.8–58.4)	41.4 (35.5–47.2)	-

ADG, Aggregated Diagnosis Group; EAC, esophageal adenocarcinoma; NA, not available.

**Fig 1:**
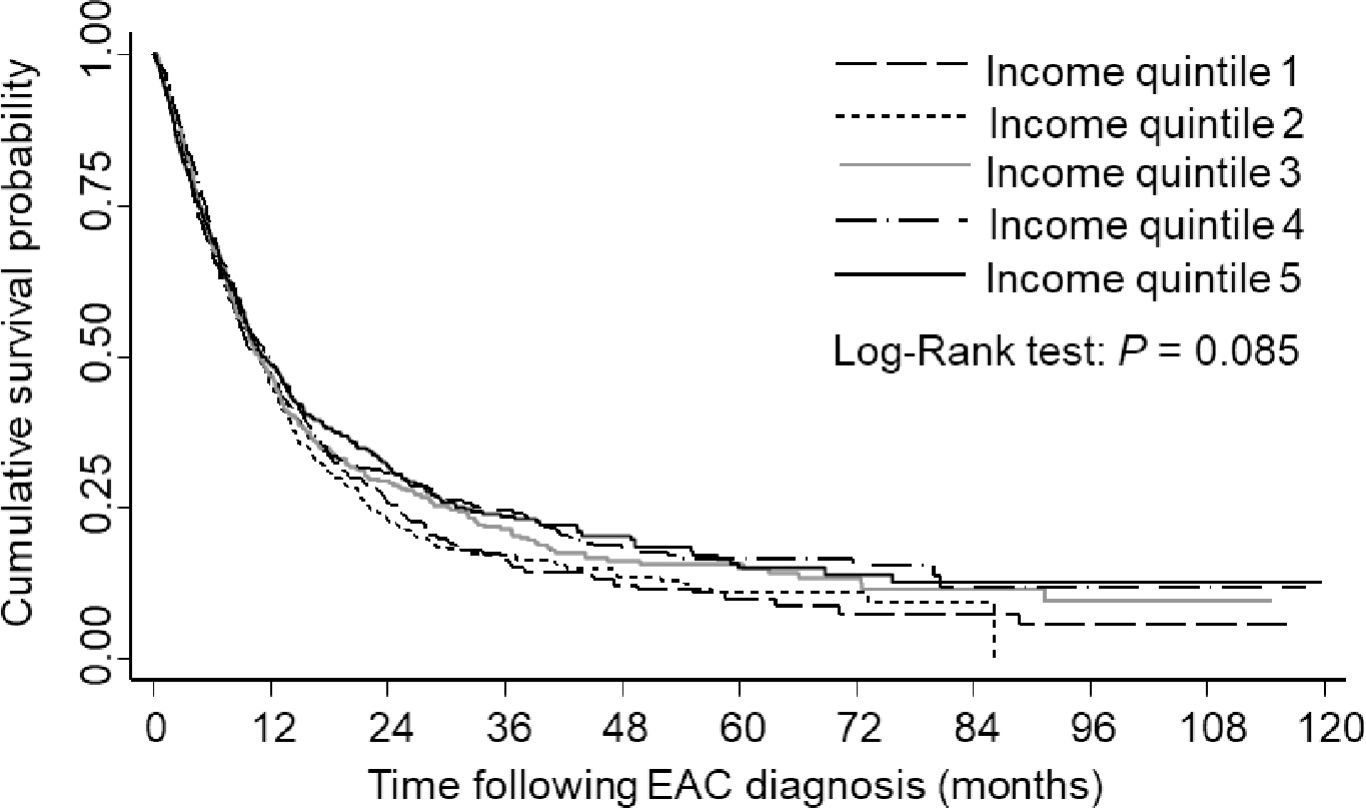
Kaplan-Meier survival estimates of people diagnosed with esophageal adenocarcinoma by socioeconomic status, 120 months follow-up time (log-rank test: *P* = 0.085). Income quintile 1, lowest socioeconomic status; Income quintile 5, highest socioeconomic status.

**Fig 2:**
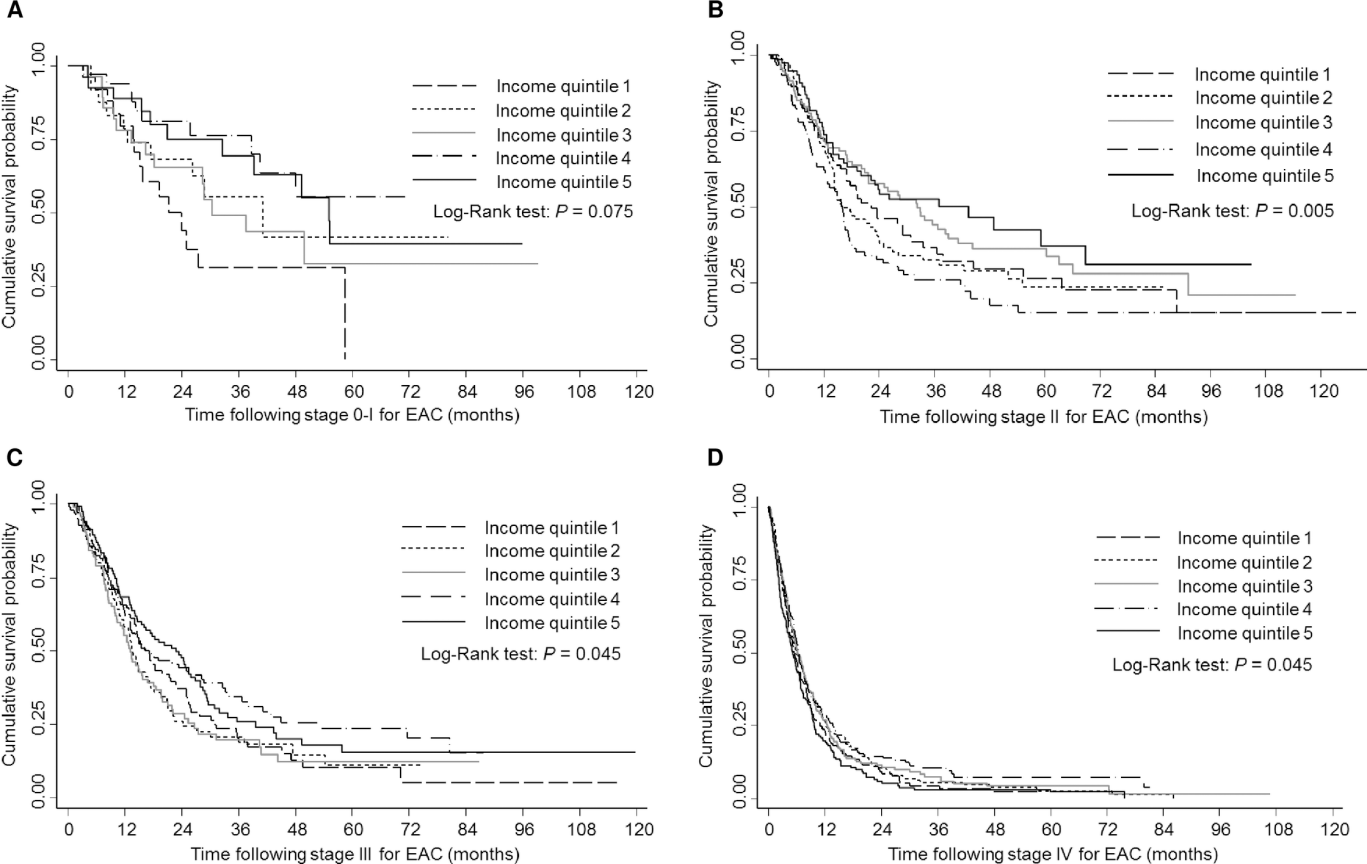
**2A-2D. Kaplan-Meier survival estimates of people according to stage at EAC diagnosis by socioeconomic status, 120 months follow-up time.** (A) Stage 0-I (log-rank test: *P* = 0.075); (B) Stage II (log-rank test: *P* = 0.005); (C) Stage III (log-rank test: *P* = 0.045); (D) Stage IV (log-rank test: *P* = 0.045). Income quintile 1, lowest socioeconomic status; Income quintile 5, highest socioeconomic status.

**Fig 3:**
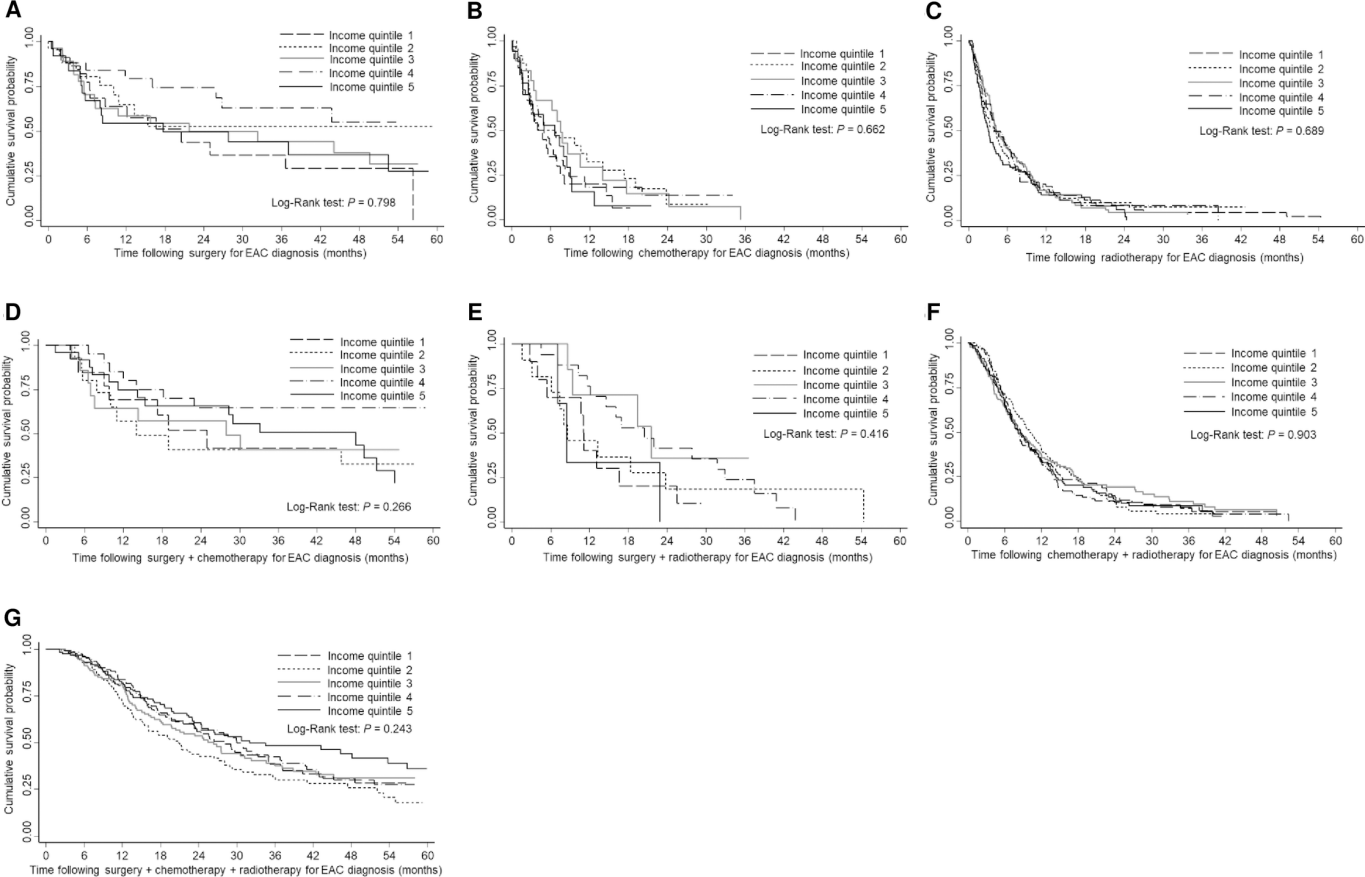
**3A-3G. Kaplan-Meier survival estimates of people who received treatment for with esophageal adenocarcinoma by socioeconomic status, 60 months follow-up time.** (A) surgery alone (log-rank test: *P* = 0.798); (B) chemotherapy alone (log-rank test: *P* = 0.662); (C) radiotherapy alone (log-rank test: *P =* 0.689); (D) surgery + chemotherapy (log-rank test: *P* = 0.266); (E) surgery + radiotherapy (log-rank test: *P* = 0.416); (F) chemotherapy + radiotherapy (log-rank test: *P* = 0.903); and (G) surgery + chemotherapy + radiotherapy (log-rank test: *P* = 0.243) Income quintile 1, lowest socioeconomic status; Income quintile 5, highest socioeconomic status.

There was no significant difference in the overall survival between the income quintiles (log-rank test *P* = 0.085), Fig 1. The 5-year survival rate seemed different between the income quintiles, with income quintile 1 having a survival rate of 9.8% after 5 years post-diagnosis of EAC compared to 15.0% for income quintile 5. When the survival curves were stratified by EAC stage, there was a significant difference in the survival between the income quintiles according to stages II (*P* = 0.005), III (*P* = 0.045), and IV (*P* = 0.045) (Fig 2A–2D). When survival curves were stratified by treatment type, there was no significant difference in survival times for the income quintiles (Fig 3A–3G).

### Association between SES and stage at EAC diagnosis, EAC treatment and health region

There was not a significant association between SES and cancer stage at EAC diagnosis, after adjusting for age, gender, residence, birth country, health region, ADG, and year of EAC diagnosis (Table 3).

**Table 3:** Odds of EAC stage among people diagnosed with esophageal adenocarcinoma by income quintile, 2003–2012.

Variable	Cancer stage at EAC diagnosis*					
	Stage II		Stage III		Stage IV	
	OR (95% CI)	*P*-value	OR (95% CI)	*P*-value	OR (95% CI)	*P*-value
Income quintile						
Q1 (lowest)	1.51 (0.57–4.02)	0.406	1.01 (0.39–2.59)	0.984	1.06 (0.43–2.62)	0.905
Q2	1.44 (0.54–3.81)	0.463	0.86 (0.33–2.21)	0.756	1.14 (0.46–2.83)	0.776
Q3	1.29 (0.50–3.32)	0.601	0.83 (0.33–2.08)	0.692	0.90 (0.37–2.18)	0.820
Q4	2.21 (0.81–6.01)	0.122	1.13 (0.42–3.00)	0.809	1.20 (0.46–3.08)	0.710
Q5 (highest)	Reference		Reference		Reference	

Total N = 1,573

*Multinomial logistic regression analysis (fully-adjusted model) overall *P*-values: income quintile (*P* = 0.558), age (*P* = 0.007), gender (*P* = 0.462), urban/rural residence (*P* = 0.280), birth country (*P* = 0.306), Ontario health region (*P* < 0.001), Aggregated Diagnosis Group (ADG) (*P* = 0.816), and year of EAC diagnosis (*P* = 0.519).

Table 4 shows the association between SES and EAC treatment received. Although there are some differences (Table 1), the statistical test for an overall association did not achieve significance.

**Table 4:** Odds of EAC treatment among people diagnosed with esophageal adenocarcinoma by income quintile, 2003–2012.

Variable	EAC treatment after diagnosis							
	Surgery alone		Chemotherapy alone		Radiotherapy alone		Surgery + Chemotherapy	
	OR (95% CI)	*P*-value	OR (95% CI)	*P*-value	OR (95% CI)	*P*-value	OR (95% CI)	*P*-value
Income quintile								
Q1 (lowest)	0.82 (0.30–2.24)	0.700	1.11 (0.43–2.91)	0.826	0.79 (0.41–1.54)	0.494	0.41 (0.13–1.27)	0.121
Q2	0.92 (0.31–2.73)	0.887	1.59 (0.61–4.18)	0.347	0.90 (0.46–1.79)	0.774	0.83 (0.27–2.53)	0.739
Q3	1.08 (0.39–2.99)	0.878	1.31 (0.48–3.57)	0.603	0.99 (0.50–1.96)	0.971	0.75 (0.24–2.33)	0.624
Q4	0.69 (0.23–2.04)	0.499	2.23 (0.86–5.80)	0.099	0.91 (0.45–1.84)	0.796	0.52 (0.17–1.65)	0.270
Q5 (highest)	Reference		Reference		Reference		Reference	
	Surgery + Radiotherapy		Chemotherapy + Radiotherapy		Surgery + Chemotherapy + Radiotherapy			
	OR (95% CI)	*P*-value	OR (95% CI)	*P*-value	OR (95% CI)	*P*-value		
Income quintile								
Q1 (lowest)	2.40 (0.54–10.70)	0.251	0.74 (0.38–1.41)	0.354	0.77 (0.36–1.64)	0.493		
Q2	4.31 (0.98–19.04)	0.054	1.15 (0.59–2.24)	0.683	1.79 (0.83–3.88)	0.137		
Q3	1.32 (0.25–7.02)	0.741	1.19 (0.61–2.33)	0.617	1.58 (0.73–3.43)	0.244		
Q4	5.15 (1.23–21.64)	**0.025**	1.05 (0.53–2.08)	0.894	0.94 (0.43–2.06)	0.871		
Q5 (highest)	Reference		Reference		Reference		

Total N = 1,573. Multinomial logistic regression analysis (fully-adjusted model) overall *P*-values: income quintile (*P* = 0.209), age (*P* < 0.001); gender (*P* = 0.665); residence (*P* = 0.178); birth country (*P* = 0.193); Ontario health region (*P* = 0.008); Aggregated Diagnosis Group (ADG) (*P* = 0.635); cancer stage at EAC diagnosis (*P* < 0.001); and year of EAC diagnosis (*P* < 0.001).

There was a significant difference between SES income quintiles among Ontario health regions when compared to Central region, except for Erie St. Clair, Waterloo Wellington, Mississauga, Toronto Central, and North West (S4 Table).

### Association between SES and EAC survival

Within the unadjusted Cox proportional-hazards model, patients with EAC in the lower three income quintiles had increased risk of mortality relative to the highest income category (Table 5); those in the lower income quintiles (Q1-Q2) experienced a 16%-17% increase in the risk of death (Q1: HR = 1.16; 95% CI, 1.00–1.36; Q2: HR = 1.17, 95% CI, 1.00–1.37). However, this association disappeared in the fully-adjusted multivariate model; there was no significant association between SES and EAC survival after controlling for age, gender, residence, birth country, health region, ADG, cancer stage, treatment, and year of diagnosis. Additionally, increased mortality risk was observed for age (*P* = 0.001), cancer stage at EAC diagnosis (*P* < 0.001), chemotherapy (*P* < 0.001), radiotherapy (*P* < 0.001), surgery plus chemotherapy (*P* < 0.001), and year of EAC diagnosis.

**Table 5:** Risk of mortality after the diagnosis of esophageal adenocarcinoma, 2003–2012: Cox proportional-hazards regression models.

Characteristics	Univariate Analysis		Multivariate Analysis	
	Hazard Ratio (95% CI)	*P*-value	Hazard Ratio (95% CI)	*P*-value
Income quintile				
1 (lowest)	1.16 (1.00–1.36)	0.056	1.03 (0.87–1.21)	0.759
2	1.17 (1.00–1.37)	**0.045**	0.94 (0.8–1.11)	0.463
3	1.06 (0.91–1.24)	0.438	0.97 (0.82–1.14)	0.712
4	1.00 (0.86–1.17)	0.958	1.05 (0.89–1.24)	0.546
5 (highest)	Reference		Reference	
Age group (years)				
<50	Reference		Reference	
50–54	0.97 (0.77–1.21)	0.771	1.19 (0.94–1.51)	0.151
55–59	0.96 (0.78–1.19)	0.721	1.16 (0.92–1.46)	0.217
60–64	0.94 (0.76–1.15)	0.531	1.29 (1.03–1.60)	**0.026**
65–69	1.02 (0.83–1.26)	0.824	1.10 (0.88–1.37)	0.423
70–74	1.19 (0.96–1.47)	0.110	1.30 (1.03–1.63)	**0.027**
75–79	1.19 (0.96–1.47)	0.115	1.25 (0.99–1.58)	0.057
80–84	1.61 (1.29–2.02)	**<0.001**	1.79 (1.38–2.31)	**<0.001**
≥85	1.46 (1.11–1.93)	**0.007**	1.48 (1.08–2.03)	**0.016**
Sex				
Male	Reference		Reference	
Female	1.12 (0.98–1.29)	0.095	1.03 (0.89–1.19)	0.688
Residence				
Rural	Reference		Reference	
Urban	1.02 (0.9–1.15)	0.789	0.92 (0.80–1.06)	0.252
Birth country				
Outside of Canada	Reference		Reference	
Canada	0.99 (0.87–1.11)	0.818	0.94 (0.82–1.06)	0.294
Ontario Health Region				
Central	Reference		Reference	
Erie St. Clair	1.35 (1.00–1.82)	0.052	1.24 (0.91–1.70)	0.180
South West	1.83 (1.41–2.36)	**<0.001**	1.34 (1.02–1.75)	**0.036**
Waterloo Wellington	1.45 (1.10–1.91)	**0.009**	1.23 (0.92–1.65)	0.155
Hamilton Niagara Haldimand Brant	1.70 (1.35–2.14)	**<0.001**	1.15 (0.90–1.46)	0.258
Central West	1.02 (0.68–1.52)	0.933	1.11 (0.74–1.68)	0.607
Mississauga	1.86 (1.32–2.63)	**0.001**	1.35 (0.94–1.95)	0.104
Toronto Central	1.22 (0.91–1.63)	0.183	1.05 (0.78–1.41)	0.760
Central East	1.25 (0.98–1.61)	0.079	1.06 (0.82–1.38)	0.649
South East	1.34 (1.03–1.74)	**0.028**	1.23 (0.93–1.63)	0.154
Champlain	1.35 (1.06–1.72)	**0.015**	1.03 (0.79–1.33)	0.844
North Simcoe	1.11 (0.82–1.50)	0.492	1.09 (0.79–1.50)	0.595
North East	1.45 (1.10–1.90)	**0.007**	1.09 (0.82–1.46)	0.558
North West	1.54 (1.11–2.15)	**0.010**	1.36 (0.95–1.96)	0.092
ADG				
0	Reference		Reference	
1–3	0.81 (0.44–1.50)	0.505	0.83 (0.43–1.60)	0.580
4–7	0.80 (0.45–1.43)	0.450	0.82 (0.44–1.51)	0.521
8–10	0.81 (0.46–1.43)	0.465	0.84 (0.46–1.55)	0.580
11+	0.77 (0.44–1.36)	0.372	0.76 (0.42–1.40)	0.380
Stage at EAC diagnosis*				
Stage 0-I	Reference		Reference	
Stage II	1.64 (1.24–2.17)	**0.001**	1.20 (0.89–1.62)	0.230
Stage III	2.23 (1.70–2.93)	**<0.001**	1.41 (1.05–1.90)	**0.024**
Stage IV	5.65 (4.35–7.35)	**<0.001**	2.66 (2.00–3.54)	**<0.001**
EAC treatment*				
Surgery (yes vs. no)	0.52 (0.45–0.59)	**<0.001**	1.14 (0.86–1.50)	0.368
Chemotherapy (yes vs. no)	1.14 (1.02–1.28)	**0.023**	1.95 (1.54–2.48)	**<0.001**
Radiotherapy (yes vs. no)	1.64 (1.49–1.82)	**<0.001**	2.28 (1.81–2.86)	**<0.001**
Surgery + chemotherapy (yes vs. no)	0.75 (0.65–0.86)	**<0.001**	1.50 (1.21–1.87)	**<0.001**
Surgery + radiotherapy (yes vs. no)	0.74 (0.42–1.30)	0.289	0.86 (0.47–1.56)	0.624
Chemotherapy + radiotherapy (yes vs. no)	1.16 (1.05–1.29)	**0.003**	1.02 (0.88–1.17)	0.813
Surgery + chemotherapy + radiotherapy (yes vs. no)	0.57 (0.51–0.65)	**<0.001**	0.92 (0.79–1.08)	0.325
Year of EAC diagnosis				
2003–2004	Reference		Reference	
2005–2006	1.09 (0.91–1.30)	0.350	1.05 (0.87–1.26)	0.630
2007–2008	1.07 (0.90–1.28)	0.444	1.13 (0.94–1.36)	0.210
2009–2010	1.00 (0.84–1.19)	0.994	1.30 (1.08–1.57)	**0.005**
2011–2012	0.73 (0.60–0.89)	**0.002**	2.26 (1.81–2.82)	**<0.001**

*Variable modeled as time-dependent covariate. ADG, Aggregated Diagnosis Group; EAC, esophageal adenocarcinoma. Univariate (unadjusted model; n = 2,115) analysis overall *P*-values: income quintile (*P* = 0.103); age (*P* < 0.001); Ontario health region (*P* < 0.001); ADG (*P* = 0.838); cancer stage at EAC diagnosis (*P* < 0.001); and year of EAC diagnosis (*P* < 0.001).

Multivariate (fully-adjusted model; n = 1,573) analysis overall *P*-values: income quintile (*P* = 0.669); age (*P* = 0.001); Ontario health region (*P* = 0.466); ADG (*P* = 0.504); cancer stage at EAC diagnosis (*P* < 0.001); and year of EAC diagnosis (*P* < 0.001).

However, including only individuals with stage 0-III cancer, and excluding those patients with more advanced-stage IV EAC, there was a significant association between SES and EAC survival after controlling for covariates (S5 Table).

### Sensitivity analysis

After multiple imputation for variables with missing data such as income quintile, urban/rural residence, birth country and cancer stage at EAC diagnosis, and after adjusting for confounding covariates, there was also no significant association between SES and stage at EAC diagnosis (S6 Table) or EAC treatment (S7 Table). Additionally, in the fully-adjusted multivariate model, patients with EAC in the lower two income quintiles had a significantly increased risk of mortality relative to the highest income category (S8 Table). When including only individuals with stage 0-III cancer, and excluding those patients with more advanced-stage IV EAC, patients with EAC in the lower four income quintiles had a significantly increased risk of mortality relative to the highest income category (S9 Table).

## Discussion

This population-based retrospective cohort study examined the effects of SES on stage of diagnosis of EAC, receiving treatment, regional variation (Ontario health region), and survival. The results indicate that individuals in lower SES categories have reduced survival compared to those in the highest income quintile, but these differences disappear after adjusting for confounders. While there is an apparent 17% increase in mortality for individuals in the lower income quintiles compared to the highest, the significance of this association disappears to almost null in the fully-adjusted regression model, after controlling for additional covariates. However, the significant association between SES and EAC survival remained when considering only those who presented with stage 0-III EAC at diagnosis. There was no significant association between SES and EAC stage at diagnosis or between income quintile and receipt of potentially curative EAC treatment.

A previous study in Ontario found no significant relationship between SES and stage at diagnosis for hepatocellular carcinoma [36]; other Canadian studies have yielded mixed results with respect to various cancers [37, 38]. Conversely, studies in the United States have found a significant relationship between SES and stage at diagnosis [39]. This may be explained by differences in health care systems, with patients in countries with privatized systems being more reluctant to seek treatment until symptoms are exacerbated.

Studies regarding the relationship between SES and survival have had conflicting results. Several studies published in Canada by Gorey et al. indicate that there is no survival gradient for SES in Ontario for type of cancer [40–43]. A study by Gorey et al. that compared the effect of SES on colon cancer and treatment in San Francisco and Toronto found that differences in SES had a more pronounced influence on survival in California compared to Ontario, with Canada's universal health care system functioning more equitably for both rich and poor [41]. Other studies including one published in 2010 by Booth et al. found that a survival gradient is in fact present between individuals of differing SES [37]. One presented explanation was the increased likelihood of higher SES individuals to receive curative treatment for hepatocellular carcinoma [19]. Corresponding to our study, a population-based study of patients with potentially resectable esophageal cancer in the Netherlands conducted by Koeter et al. found that surgical resection occurred less often among less well-off patients; however, survival was not significantly affected by SES [44].

A previous study conducted by Tinmouth et al. in 2011, found that compared to the Central region, people in the North West Ontario health region were 6.5 times as likely to contract EAC [45]. Possible explanations for this phenomenon include ethnic variation and suboptimal treatment center placement. Our study identified differences in SES quintiles by health region, however, the adjusted proportional-hazards model findings observed no association between health region and EAC survival. Differences in the health region distribution of EAC cases may also be explained by physician supply. A previous study conducted in Ontario found that access to regional primary care physicians was significantly correlated with health outcomes [46, 47]. These results indicate a need to further research the health effects of physician supply and the efficacy of current health care system distribution patterns.

Our study was large and population-based. It has some limitations, however. The study design was retrospective and so cannot establish causation. SES was an ecological variable and may not be fully indicative of true individual-level SES. Median neighborhood household income also fails to account for several other important determinants of SES including social status, employment type, and social capital. We also could not account for several primary risk factors for EAC, including GERD symptoms, Barrett’s esophagus, tobacco smoking, race or ethnicity, and alcohol consumption, due to a lack of data. Population distributions of these risk factors may accompany disparities in SES. European studies have indicated that lower SES is associated with worsened occurrence of GERD symptoms [48, 49]. Conflicting results have been reported regarding Barrett's esophagus; a study in the United States found that greater education is associated with a reduced likelihood of Barrett's esophagus [50], while a United Kingdom study found that increased Barrett's esophagus risk accompanies increased SES [9]. In Canada, increased tobacco use is observed among those at lower SES levels [51], while United Kingdom researchers posit that alcohol use leads to more deleterious health impacts among populations experiencing greater social deprivation [52]. We could not assess screening for EAC (endoscopy and biopsy), prior Barrett’s esophagus diagnosis, or prior cancer diagnosis. EAC patients with a prior Barrett’s esophagus diagnosis are commonly diagnosed with earlier stage disease and have improved survival compared with EAC patients with no prior Barrett’s esophagus diagnosis [53]. We were not able to discern whether patients did not receive treatment because they were not offered it, had poor functional status, declined it, or experienced other barriers [54]. Lastly, stage at diagnosis was missing for a lot of patients and they were excluded from the primary analysis. Statistically, multiple imputation is an established method to deal with replacing each missing value with a set of plausible values to ensure the results are unbiased and capture the appropriate degree of precision.

## Conclusion

We saw a direct association between SES and EAC survival in Ontario, but this could be explained by patient-level confounders, including receipt of treatment. This indicates that even in our universal health care system there are inequities that affect survival. Further work needs to be done to identify the reasons for any barriers to treatment and find ways to overcome these barriers.

## Supporting information

S1 Fig Selection criteria for the study sample. (TIF)

S1 Table Codes used to define cases of esophageal adenocarcinoma. (DOCX)

S2 Table Fee codes used to define types of treatment for esophageal adenocarcinoma. (DOCX)

S3 Table Sociodemographic and clinical characteristics of people diagnosed with esophageal adenocarcinoma, 1993–2012. (DOCX)

S4 Table Odds of Ontario health region among people diagnosed with esophageal adenocarcinoma by income quintile, 2003–2012. (DOCX)

S5 Table Risk of mortality after the diagnosis of esophageal adenocarcinoma, 2003–2012: Cox proportional-hazards regression models: excluding advanced-stage IV. (DOCX)

S6 Table Odds of EAC stage among people diagnosed with esophageal adenocarcinoma by income quintile, 1993–2012: Multiple imputation method. (DOCX)

S7 Table Odds of EAC treatment among people diagnosed with esophageal adenocarcinoma by income quintile, 1993–2012: Multiple imputation method. (DOCX)

S8 Table Risk of mortality after the diagnosis of esophageal adenocarcinoma, 1993–2012: Cox proportional-hazards regression models: Multiple imputation method. (DOCX)

S9 Table Risk of mortality after the diagnosis of esophageal adenocarcinoma, 1993–2012: Cox proportional-hazards regression models: Multiple imputation, excluding advanced-stage IV. (DOCX)

## Abbreviations

EAC: esophageal adenocarcinoma
GERD: gastroesophageal reflux disease
SES: socioeconomic status
OCR: Ontario Cancer Registry
ICD-9: International Statistical Classification of Diseases and Related Health Problems, 9th Revision
ICD-O-3: International Classification of Diseases for Oncology, Third Edition
ADG: aggregated diagnosis groups
ACG: Adjusted Clinical Groups
IQR: interquartile range
CI: confidence interval
