# Supplementary material for: Effects of socioeconomic status on esophageal adenocarcinoma stage at diagnosis, receipt of treatment, and survival: A population-based cohort study
Source: PLoS One. 2017 Oct 11;12(10):e0186350. doi: 10.1371/journal.pone.0186350 (PMC5636169; doi:10.1371/journal.pone.0186350)
Supplement: S1 Table — (DOCX) [file pone.0186350.s002.docx]

**S1 Table. Codes used to define cases of esophageal adenocarcinoma**

| **Data elements** | **Ontario Cancer Registry (OCR)** |
| --- | --- |
| ICD-9 | 150 |
| Histology ICD-O-3 | 8140, 8141, 8143, 8144, 8145, 8147, 8200, 8201, 8255, 8260, 8261, 8262, 8263, 8430, 8480, 8481, 8560, 8562, 8570, 8571, 8572, 8573, 8574, 8575 |

ICD-9, International Statistical Classification of Diseases and Related Health Problems, 9th Revision; ICD-O-3, International Classification of Diseases for Oncology, Third Edition.
