# Supplementary material for: Effects of socioeconomic status on esophageal adenocarcinoma stage at diagnosis, receipt of treatment, and survival: A population-based cohort study
Source: PLoS One. 2017 Oct 11;12(10):e0186350. doi: 10.1371/journal.pone.0186350 (PMC5636169; doi:10.1371/journal.pone.0186350)
Supplement: S2 Table — (DOCX) [file pone.0186350.s003.docx]

**S2 Table. Fee codes used to define types of treatment for esophageal adenocarcinoma**

| **Treatment** | **Code Type** | | **References** |
| --- | --- | --- | --- |
|  | **OHIP** | **CIHI-DAD & NACRS** |  |
| Surgical resection | S089, S090, S123, S125, S128 | 1NA55, 1NA76, 1NA77, 1NA87, 1NA88, 1NA89, 1NA90, 1NA91, 1NA92 | [[1](#_ENREF_1), [2](#_ENREF_2)] |
| Chemotherapy | G339, G345, G359, G281, G381, G382, G388 | 1NA35, 1ZZ35 | [[2](#_ENREF_2), [3](#_ENREF_3)] |
| Radiotherapy | X310, X311, X312, X313 | 1NA26, 1NA27 | [[2](#_ENREF_2), [4](#_ENREF_4)] |
| Palliative procedures |  |  | [[2](#_ENREF_2), [5](#_ENREF_5)] |
| Esophageal dilation | E696, E698, Z523, Z525, Z529 | 1NA50, 1NA53 |  |
| Drainage |  | 1NA52 |  |
| esophageal stenting | E629, S082, S083 |  |  |
| Laser debulking of tumor | E692, E695 | 1NA59 |  |
| Palliative care | E083, G511, G512, C882, C982, K023, C945 |  |  |

OHIP, Ontario Health Insurance Plan; CIHI, Canadian Institute for Health Information; DAD, Discharge Abstract Database; NACRS, National Ambulatory Care Reporting System

**References**

1. Digestive System Surgical Procedures. Available at: <http://www.health.gov.on.ca/en/pro/programs/ohip/sob/physserv/s_digest.pdf>. Accessed March 9, 2017.

2. Canadian Classification of Health Interventions. Volume Four — Alphabetical Index. Canadian Institute for Health Information 2012. Available at: https://[www.cihi.ca/en/cci_volume_four_2012_en.pdf](http://www.cihi.ca/en/cci_volume_four_2012_en.pdf). Accessed March 9, 2017.

3. Diagnostic and Therapeutic Procedures. Available at: <http://www.health.gov.on.ca/en/pro/programs/ohip/sob/physserv/j_diagth.pdf>. Accessed March 9, 2017.

4. Radiation Oncology. Available at: <http://health.gov.on.ca/en/pro/programs/ohip/sob/physserv/c_radiat.pdf>. Accessed March 9, 2017.

5. Guide to OHIP Billing for Palliative Care Services. Last Updated: February 23, 2015. Available at: https://hqic.ca/Pages/HqicPrivacyDocs.aspx?docNm=palliative-care-billing.pdf. Accessed March 9, 2017.
