# Supplementary material for: Effects of socioeconomic status on esophageal adenocarcinoma stage at diagnosis, receipt of treatment, and survival: A population-based cohort study
Source: PLoS One. 2017 Oct 11;12(10):e0186350. doi: 10.1371/journal.pone.0186350 (PMC5636169; doi:10.1371/journal.pone.0186350)
Supplement: S3 Table — (DOCX) [file pone.0186350.s004.docx]

**S3 Table. Sociodemographic and clinical characteristics of people diagnosed with esophageal adenocarcinoma, 1993-2012**

| **Variable** |  | **Year of EAC diagnosis** | | | |  |
| --- | --- | --- | --- | --- | --- | --- |
|  | **TOTAL** | **1993-1997** | **1998-2002** | **2003-2007** | **2008-2012** | ***P*-value** |
|  | **N (%)** | **N (%)** | **N (%)** | **N (%)** | **N (%)** |  |
| Total N (%) | 5382 (100) | 859 (16.0) | 1149 (21.4) | 1484 (27.6) | 1890 (35.1) |  |
| Income quintile |  |  |  |  |  |  |
| Q1 (lowest) | 1092 (20.3) | 183 (21.3) | 237 (20.6) | 288 (19.4) | 384 (20.3) |  |
| Q2 | 1108 (20.6) | 159 (18.5) | 244 (21.2) | 297 (20.0) | 408 (21.6) |  |
| Q3 | 1084 (20.1) | 182 (21.2) | 238 (20.7) | 314 (21.2) | 350 (18.5) |  |
| Q4 | 1054 (19.6) | 163 (19.0) | 204 (17.8) | 296 (20.0) | 391 (20.7) |  |
| Q5 (highest) | 1024 (19.0) | 166 (19.3) | 225 (19.6) | 282 (19.0) | 351 (18.6) |  |
| Missing | 20 (0.4) | 6 (0.7) | ‒ (0.1) | 7 (0.5) | 6 (0.3) | 0.290 |
| Age group (years) |  |  |  |  |  |  |
| <50 | 455 (8.5) | 78 (9.1) | 115 (10.0) | 136 (9.2) | 126 (6.7) |  |
| 50-54 | 458 (8.5) | 65 (7.6) | 93 (8.1) | 135 (9.1) | 165 (8.7) |  |
| 55-59 | 585 (10.9) | 78 (9.1) | 122 (10.6) | 156 (10.5) | 229 (12.1) |  |
| 60-64 | 740 (13.8) | 101 (11.8) | 122 (10.6) | 210 (14.2) | 307 (16.2) |  |
| 65-69 | 807 (15.0) | 136 (15.8) | 186 (16.2) | 195 (13.1) | 290 (15.3) |  |
| 70-74 | 793 (14.7) | 155 (18.0) | 179 (15.6) | 210 (14.2) | 249 (13.2) |  |
| 75-79 | 690 (12.8) | 118 (13.7) | 155 (13.5) | 212 (14.3) | 205 (10.9) |  |
| 80-84 | 508 (9.4) | 84 (9.8) | 105 (9.1) | 140 (9.4) | 179 (9.5) |  |
| >85 | 346 (6.4) | 44 (5.1) | 72 (6.3) | 90 (6.1) | 140 (7.4) | **<0.001** |
| Sex |  |  |  |  |  |  |
| Male | 4520 (84.0) | 716 (83.4) | 976 (84.9) | 1231 (83.0) | 1597 (84.5) |  |
| Female | 862 (16.0) | 143 (16.7) | 173 (15.1) | 253 (17.1) | 293 (15.5) | 0.460 |
| Residence |  |  |  |  |  |  |
| Urban | 4353 (80.9) | 687 (80) | 924 (80.4) | 1219 (82.1) | 1523 (80.6) |  |
| Rural | 1026 (19.1) | 169 (19.7) | 225 (19.6) | 265 (17.9) | 367 (19.4) |  |
| Missing | ‒ (0.1) | ‒ (0.4) | 0 | 0 | 0 | **0.007** |
| Birth country |  |  |  |  |  |  |
| Canada | 3337 (62.0) | 618 (71.9) | 798 (69.5) | 984 (66.3) | 937 (49.6) |  |
| Outside Canada | 1085 (20.2) | 206 (24) | 280 (24.4) | 322 (21.7) | 277 (14.7) |  |
| Missing | 960 (17.8) | 35 (4.1) | 71 (6.2) | 178 (12.0) | 676 (35.8) | **<0.001** |

S3 Table continued on the following page

**S3 Table. Sociodemographic and clinical characteristics of people diagnosed with esophageal adenocarcinoma, 1993-2012 (continued)**

| **Variable** |  | **Year of EAC diagnosis** | | | |  |
| --- | --- | --- | --- | --- | --- | --- |
|  | **TOTAL** | **1993-1997** | **1998-2002** | **2003-2007** | **2008-2012** | ***P*-value** |
|  | **N (%)** | **N (%)** | **N (%)** | **N (%)** | **N (%)** |  |
| Ontario Health Regions |  |  |  |  |  |  |
| Erie St. Clair | 285 (5.3) | 46 (5.4) | 63 (5.5) | 75 (5.1) | 101 (5.3) |  |
| South West | 502 (9.3) | 91 (10.6) | 104 (9.1) | 115 (7.8) | 192 (10.2) |  |
| Waterloo Wellington | 311 (5.8) | 57 (6.6) | 58 (5.1) | 73 (4.9) | 123 (6.5) |  |
| Hamilton Niagara Haldimand Brant | 767 (14.3) | 101 (11.8) | 179 (15.6) | 221 (14.9) | 266 (14.1) |  |
| Central West | 183 (3.4) | 31 (3.6) | 44 (3.8) | 55 (3.7) | 53 (2.8) |  |
| Mississauga | 239 (4.4) | 38 (4.4) | 44 (3.8) | 65 (4.4) | 92 (4.9) |  |
| Toronto Central | 346 (6.4) | 61 (7.1) | 63 (5.5) | 99 (6.7) | 123 (6.5) |  |
| Central | 385 (7.2) | 64 (7.5) | 76 (6.6) | 122 (8.2) | 123 (6.5) |  |
| Central East | 591 (11.0) | 96 (11.2) | 121 (10.5) | 163 (11.0) | 211 (11.2) |  |
| South East | 421 (7.8) | 73 (8.5) | 105 (9.1) | 113 (7.6) | 130 (6.9) |  |
| Champlain | 582 (10.8) | 87 (10.1) | 125 (10.9) | 166 (11.2) | 204 (10.8) |  |
| North Simcoe | 257 (4.8) | 33 (3.8) | 52 (4.5) | 81 (5.5) | 91 (4.8) |  |
| North East | 371 (6.9) | 61 (7.1) | 85 (7.4) | 95 (6.4) | 130 (6.9) |  |
| North West | 142 (2.6) | 20 (2.3) | 30 (2.6) | 41 (2.8) | 51 (2.7) | 0.398 |
| ADG |  |  |  |  |  |  |
| 0 | 36 (0.7) | 11 (1.3) | 8 (0.7) | 10 (0.7) | 7 (0.4) |  |
| 1-3 | 253 (4.7) | 63 (7.3) | 61 (5.3) | 61 (4.1) | 68 (3.6) |  |
| 4-7 | 1067 (19.8) | 196 (22.8) | 252 (21.9) | 270 (18.2) | 349 (18.5) |  |
| 8-10 | 1420 (26.4) | 218 (25.4) | 302 (26.3) | 362 (24.4) | 538 (28.5) |  |
| 11+ | 2606 (48.4) | 371 (43.2) | 526 (45.8) | 781 (52.6) | 928 (49.1) | **<0.001** |
| Stage at EAC diagnosis |  |  |  |  |  |  |
| Stage 0-I | 145 (2.7) | 0 | 0 | 38 (2.6) | 107 (5.7) |  |
| Stage II | 445 (8.3) | 0 | 0 | 161 (10.9) | 284 (15.0) |  |
| Stage III | 515 (9.6) | 0 | 0 | 200 (13.5) | 315 (16.7) |  |
| Stage IV | 1020 (19.0) | 0 | 0 | 438 (29.5) | 582 (30.8) |  |
| Unknown | 3257 (60.5) | 859 (100) | 1149 (100) | 647 (43.6) | 602 (31.9) | **<0.001** |

S3 Table continued on the following page

**S3 Table. Sociodemographic and clinical characteristics of people diagnosed with esophageal adenocarcinoma, 1993-2012 (continued)**

| **Variable** |  | **Year of EAC diagnosis** | | | |  |
| --- | --- | --- | --- | --- | --- | --- |
|  | **TOTAL** | **1993-1997** | **1998-2002** | **2003-2007** | **2008-2012** | ***P*-value** |
|  | **N (%)** | **N (%)** | **N (%)** | **N (%)** | **N (%)** |  |
| EAC treatment |  |  |  |  |  |  |
| Surgery alone | 815 (15.1) | 246 (28.6) | 228 (19.8) | 167 (11.3) | 174 (9.2) |  |
| Chemotherapy alone | 522 (9.7) | 10 (1.2) | 90 (7.8) | 230 (15.5) | 192 (10.2) |  |
| Radiotherapy alone | 450 (8.4) | 7 (0.8) | 11 (1.0) | 151 (10.2) | 281 (14.9) |  |
| Surgery + chemotherapy | 380 (7.1) | 14 (1.6) | 70 (6.1) | 158 (10.7) | 138 (7.3) |  |
| Surgery + radiotherapy | 48 (0.9) | 0 | 4 (0.4) | 20 (1.4) | 24 (1.3) |  |
| Chemotherapy + radiotherapy | 613 (11.4) | 2 (0.2) | 31 (2.7) | 238 (16.0) | 342 (18.1) |  |
| Surgery + chemotherapy + radiotherapy | 537 (10.0) | 1 (0.1) | 13 (1.1) | 177 (11.9) | 346 (18.3) |  |
| No treatment | 2017 (37.5) | 579 (67.4) | 702 (61.1) | 343 (23.1) | 393 (20.8) | **<0.001** |
| Palliative care | 3398 (63.1) | 415 (48.3) | 633 (55.1) | 942 (63.5) | 1408 (74.5) | **<0.001** |
| Deaths | 4515 (83.9) | 833 (97.0) | 1072 (93.3) | 1318 (88.8) | 1292 (68.4) | **<0.001** |

“‒“, counts less than 6 are suppressed. ADG, Aggregated Diagnosis Group; EAC, esophageal adenocarcinoma.

*P*-value comparisons are across variable levels and include missing values.
