# Supplementary material for: Effects of socioeconomic status on esophageal adenocarcinoma stage at diagnosis, receipt of treatment, and survival: A population-based cohort study
Source: PLoS One. 2017 Oct 11;12(10):e0186350. doi: 10.1371/journal.pone.0186350 (PMC5636169; doi:10.1371/journal.pone.0186350)
Supplement: S4 Table — (DOCX) [file pone.0186350.s005.docx]

**S4 Table. Odds of Ontario health region among people diagnosed with esophageal adenocarcinoma by income quintile, 2003-2012**

| **Variable** | **Ontario health region^*^** | | | | | | | | | | | | | |
| --- | --- | --- | --- | --- | --- | --- | --- | --- | --- | --- | --- | --- | --- | --- |
|  | **Erie St. Clair** | |  | **South West** | |  | **Waterloo Wellington** | |  | **Hamilton Niagara Haldimand Brant** | |  | **Central West** | |
|  | **OR (95% CI)** | ***P*-value** |  | **OR (95% CI)** | ***P*-value** |  | **OR (95% CI)** | ***P*-value** |  | **OR (95% CI)** | ***P*-value** |  | **OR (95% CI)** | ***P*-value** |
| Income quintile |  |  |  |  |  |  |  |  |  |  |  |  |  |  |
| Q1 (lowest) | 2.01 (0.70-5.77) | 0.196 |  | 4.24 (1.65-10.93) | **0.003** |  | 1.46 (0.56-3.79) | 0.435 |  | 3.89 (1.75-8.68) | **0.001** |  | 3.95 (0.73-21.25) | 0.110 |
| Q2 | 1.81 (0.68-4.82) | 0.237 |  | 4.18 (1.75-10.03) | **0.001** |  | 1.80 (0.76-4.24) | 0.178 |  | 2.69 (1.27-5.71) | **0.010** |  | 4.33 (0.94-19.86) | 0.059 |
| Q3 | 2.67 (0.98-7.28) | 0.055 |  | 4.36 (1.71-11.16) | **0.002** |  | 1.24 (0.47-3.27) | 0.659 |  | 3.21 (1.44-7.16) | **0.004** |  | 4.07 (0.80-20.65) | 0.090 |
| Q4 | 0.94 (0.36-2.40) | 0.891 |  | 1.5 (0.64-3.51) | 0.346 |  | 0.61 (0.26-1.45) | 0.264 |  | 1.38 (0.69-2.75) | 0.363 |  | 4.28 (1.04-17.65) | **0.044** |
| Q5 (highest) | Reference |  |  | Reference |  |  | Reference |  |  | Reference |  |  | Reference |  |
|  |  |  |  |  |  |  |  |  |  |  |  |  |  |  |
|  | **Mississauga** | |  | **Toronto Central** | |  | **Central East** | |  | **South East** | |  | **Champlain** | |
|  | **OR (95% CI)** | ***P*-value** |  | **OR (95% CI)** | ***P*-value** |  | **OR (95% CI)** | ***P*-value** |  | **OR (95% CI)** | ***P*-value** |  | **OR (95% CI)** | ***P*-value** |
| Income quintile |  |  |  |  |  |  |  |  |  |  |  |  |  |  |
| Q1 (lowest) | 0.81 (0.23-2.86) | 0.738 |  | 1.65 (0.63-4.36) | 0.309 |  | 2.97 (1.22-7.23) | **0.016** |  | 8.20 (3.07-21.92) | **<0.001** |  | 2.16 (0.91-5.09) | 0.079 |
| Q2 | 0.23 (0.05-1.20) | 0.082 |  | 1.10 (0.43-2.82) | 0.851 |  | 2.23 (0.97-5.14) | **0.060** |  | 4.86 (1.87-12.60) | **0.001** |  | 1.78 (0.8-3.94) | 0.155 |
| Q3 | 1.40 (0.46-4.28) | 0.552 |  | 1.85 (0.72-4.73) | 0.201 |  | 3.46 (1.45-8.25) | **0.005** |  | 5.26 (1.93-14.32) | **0.001** |  | 2.59 (1.12-6.00) | **0.027** |
| Q4 | 0.98 (0.38-2.55) | 0.970 |  | 0.67 (0.28-1.60) | 0.365 |  | 1.62 (0.76-3.46) | 0.214 |  | 1.81 (0.72-4.58) | 0.207 |  | 0.91 (0.43-1.91) | 0.800 |
| Q5 (highest) | Reference |  |  | Reference |  |  | Reference |  |  | Reference |  |  | Reference |  |

S4 Table continued on the following page

**S4 Table. Odds of Ontario health region among people diagnosed with esophageal adenocarcinoma by income quintile, 2003-2012 (continued)**

| **Variable** | **Ontario health region^*^** | | | | | | | |
| --- | --- | --- | --- | --- | --- | --- | --- | --- |
|  | **North Simcoe** | |  | **North East** | |  | **North West** | |
|  | **OR (95% CI)** | ***P*-value** |  | **OR (95% CI)** | ***P*-value** |  | **OR (95% CI)** | ***P*-value** |
| Income quintile |  |  |  |  |  |  |  |  |
| Q1 (lowest) | 2.93 (1.07-8.00) | **0.036** |  | 9.44 (3.38-26.34) | **<0.001** |  | 1.80 (0.54-6.02) | 0.337 |
| Q2 | 0.66 (0.21-2.11) | 0.481 |  | 5.69 (2.11-15.38) | **0.001** |  | 2.39 (0.82-6.97) | 0.110 |
| Q3 | 3.02 (1.12-8.10) | **0.028** |  | 4.69 (1.61-13.68) | **0.005** |  | 2.12 (0.66-6.76) | 0.205 |
| Q4 | 0.82 (0.31-2.13) | 0.678 |  | 1.75 (0.65-4.73) | 0.272 |  | 0.92 (0.31-2.77) | 0.885 |
| Q5 (highest) | Reference |  |  | Reference |  |  | Reference |  |

Total N = 1,573

^*^Multinomial logistic regression analysis (fully-adjusted model; ‘Central’ health region = reference) overall *P*-values: income quintile (*P* < 0.001); age (*P* = 0.138); gender (*P* = 0.023); residence (*P* < 0.001); birth country (*P* < 0.001); Aggregated Diagnosis Group (ADG) (*P* = 0.535); cancer stage at EAC diagnosis (*P* < 0.001); EAC treatment (*P* = 0.026); and year of EAC diagnosis (*P* = 0.087).
