# Supplementary material for: Effects of socioeconomic status on esophageal adenocarcinoma stage at diagnosis, receipt of treatment, and survival: A population-based cohort study
Source: PLoS One. 2017 Oct 11;12(10):e0186350. doi: 10.1371/journal.pone.0186350 (PMC5636169; doi:10.1371/journal.pone.0186350)
Supplement: S5 Table — (DOCX) [file pone.0186350.s006.docx]

**S5 Table**. **Risk of mortality after the diagnosis of esophageal adenocarcinoma, 2003-2012: Cox proportional-hazards regression models: excluding advanced-stage IV**

| Characteristics | Univariate Analysis | |  | Multivariate Analysis | |
| --- | --- | --- | --- | --- | --- |
|  | Hazard Ratio (95% CI) | *P*-value |  | Hazard Ratio (95% CI) | *P*-value |
| Income quintile |  |  |  |  |  |
| 1 (lowest) | 1.35 (1.06-1.7) | **0.014** |  | 1.22 (0.94-1.57) | 0.129 |
| 2 | 1.37 (1.08-1.75) | **0.010** |  | 1.6 (1.22-2.1) | **0.001** |
| 3 | 1.21 (0.96-1.54) | 0.109 |  | 1.34 (1.04-1.74) | **0.026** |
| 4 | 1.22 (0.96-1.54) | 0.099 |  | 1.43 (1.11-1.83) | **0.006** |
| 5 (highest) | Reference |  |  | Reference |  |
| Age group (years) |  |  |  |  |  |
| <50 | Reference |  |  | Reference |  |
| 50-54 | 0.92 (0.64-1.32) | 0.648 |  | 1.01 (0.69-1.48) | 0.961 |
| 55-59 | 0.92 (0.65-1.3) | 0.639 |  | 0.85 (0.59-1.22) | 0.369 |
| 60-64 | 0.77 (0.55-1.08) | 0.134 |  | 1.05 (0.74-1.5) | 0.786 |
| 65-69 | 1.05 (0.76-1.46) | 0.767 |  | 0.95 (0.67-1.35) | 0.777 |
| 70-74 | 1.17 (0.84-1.62) | 0.359 |  | 0.92 (0.64-1.31) | 0.633 |
| 75-79 | 1.33 (0.95-1.85) | 0.098 |  | 1.33 (0.94-1.9) | 0.112 |
| 80-84 | 1.84 (1.3-2.61) | **0.001** |  | 1.37 (0.91-2.07) | 0.130 |
| >85 | 2.15 (1.47-3.16) | **<0.001** |  | 1.2 (0.74-1.93) | 0.466 |
| Sex |  |  |  |  |  |
| Male | Reference |  |  | Reference |  |
| Female | 1.28 (1.05-1.57) | **0.015** |  | 1.11 (0.89-1.4) | 0.347 |
| Residence |  |  |  |  |  |
| Rural | Reference |  |  | Reference |  |
| Urban | 1.02 (0.85-1.23) | 0.814 |  | 0.82 (0.66-1.03) | 0.088 |
| Birth country |  |  |  |  |  |
| Outside of Canada | Reference |  |  | Reference |  |
| Canada | 0.95 (0.8-1.14) | 0.606 |  | 1.02 (0.84-1.24) | 0.840 |

S5 Table continued on the following page

**S5 Table**. **Risk of mortality after the diagnosis of esophageal adenocarcinoma, 2003-2012: Cox proportional-hazards regression models: excluding advanced-stage IV** **(continued)**

| Characteristics | Univariate Analysis | |  | Multivariate Analysis | |
| --- | --- | --- | --- | --- | --- |
|  | Hazard Ratio (95% CI) | *P*-value |  | Hazard Ratio (95% CI) | *P*-value |
| Ontario Health Region |  |  |  |  |  |
| Central | Reference |  |  | Reference |  |
| Erie St. Clair | 1.34 (0.85-2.11) | 0.211 |  | 1.26 (0.77-2.06) | 0.360 |
| South West | 1.52 (1.01-2.3) | **0.046** |  | 1.08 (0.68-1.73) | 0.740 |
| Waterloo Wellington | 1.69 (1.14-2.5) | **0.010** |  | 1.41 (0.93-2.15) | 0.110 |
| Hamilton Niagara Haldimand Brant | 1.58 (1.12-2.22) | **0.009** |  | 1.18 (0.82-1.71) | 0.379 |
| Central West | 0.84 (0.45-1.55) | 0.575 |  | 0.78 (0.41-1.49) | 0.452 |
| Mississauga | 1.7 (1-2.9) | 0.052 |  | 1.66 (0.94-2.94) | 0.081 |
| Toronto Central | 1.58 (1.07-2.34) | **0.022** |  | 1.06 (0.7-1.62) | 0.771 |
| Central East | 1.28 (0.89-1.85) | 0.186 |  | 1.08 (0.73-1.59) | 0.714 |
| South East | 1.21 (0.83-1.77) | 0.333 |  | 0.9 (0.59-1.39) | 0.647 |
| Champlain | 1.1 (0.76-1.59) | 0.634 |  | 1 (0.67-1.5) | 0.999 |
| North Simcoe | 1.17 (0.77-1.79) | 0.459 |  | 1.19 (0.75-1.89) | 0.463 |
| North East | 1.64 (1.11-2.43) | **0.013** |  | 1.1 (0.72-1.7) | 0.654 |
| North West | 1.53 (0.95-2.48) | 0.083 |  | 1.37 (0.81-2.32) | 0.239 |
| ADG |  |  |  |  |  |
| 0 | Reference |  |  | Reference |  |
| 1-3 | 1.55 (0.37-6.57) | 0.553 |  | 0.13 (0.03-0.62) | **0.010** |
| 4-7 | 1.59 (0.39-6.44) | 0.516 |  | 0.11 (0.02-0.5) | **0.004** |
| 8-10 | 1.66 (0.41-6.71) | 0.477 |  | 0.12 (0.03-0.56) | **0.007** |
| 11+ | 1.73 (0.43-6.96) | 0.440 |  | 0.14 (0.03-0.61) | **0.010** |
| Stage at EAC diagnosis |  |  |  |  |  |
| Stage 0-I | Reference |  |  | Reference |  |
| Stage II | 1.66 (1.26-2.20) | **<0.001** |  | 1.21 (0.89-1.64) | 0.229 |
| Stage III | 2.32 (1.77-3.05) | **<0.001** |  | 1.57 (1.15-2.14) | **0.005** |

S5 Table continued on the following page

**S5 Table**. **Risk of mortality after the diagnosis of esophageal adenocarcinoma, 2003-2012: Cox proportional-hazards regression models: excluding advanced-stage IV** **(continued)**

| Characteristics | Univariate Analysis | |  | Multivariate Analysis | |
| --- | --- | --- | --- | --- | --- |
|  | Hazard Ratio (95% CI) | *P*-value |  | Hazard Ratio (95% CI) | *P*-value |
| EAC treatment^*^ |  |  |  |  |  |
| Surgery (yes vs. no) | 0.67 (0.57-0.79) | **<0.001** |  | 0.99 (0.6-1.63) | 0.957 |
| Chemotherapy (yes vs. no) | 1.09 (0.91-1.31) | 0.361 |  | 1.65 (0.99-2.74) | 0.054 |
| Radiotherapy (yes vs. no) | 1.48 (1.27-1.71) | **<0.001** |  | 2.03 (1.25-3.29) | **0.004** |
| Surgery + chemotherapy (yes vs. no) | 0.93 (0.78-1.11) | 0.429 |  | 1.53 (1.15-2.04) | **0.004** |
| Surgery + radiotherapy (yes vs. no) | 1.04 (0.56-1.94) | 0.910 |  | 0.69 (0.35-1.38) | 0.294 |
| Chemotherapy + radiotherapy (yes vs. no) | 1.11 (0.96-1.29) | 0.157 |  | 1.12 (0.85-1.47) | 0.442 |
| Surgery + chemotherapy + radiotherapy (yes vs. no) | 0.76 (0.65-0.9) | **0.001** |  | 0.84 (0.68-1.03) | 0.092 |
| Year of EAC diagnosis |  |  |  |  |  |
| 2003-2004 | Reference |  |  | Reference |  |
| 2005-2006 | 1.25 (0.95-1.64) | 0.109 |  | 1.19 (0.89-1.59) | 0.243 |
| 2007-2008 | 1.17 (0.9-1.54) | 0.248 |  | 1.38 (1.04-1.85) | **0.028** |
| 2009-2010 | 1.14 (0.87-1.48) | 0.347 |  | 1.8 (1.34-2.42) | **<0.001** |
| 2011-2012 | 0.79 (0.58-1.08) | 0.133 |  | 3.43 (2.41-4.9) | **<0.001** |

^*^Variable modeled as time-dependent covariate. ADG, Aggregated Diagnosis Group; EAC, esophageal adenocarcinoma.

Univariate (unadjusted model; n = 1,104) analysis overall *P*-values: income quintile (*P* = 0.077); age (*P* < 0.001); Ontario health region (*P* = 0.046); ADG (*P* = 0.837); cancer stage at EAC diagnosis (*P* < 0.001); and year of EAC diagnosis (*P* = 0.008).

Multivariate (fully-adjusted model; n = 683) analysis overall *P*-values: income quintile (*P* = 0.008); age (*P* = 0.108); Ontario health region (*P* = 0.538); ADG (*P* = 0.031); cancer stage at EAC diagnosis (*P* = 0.002); and year of EAC diagnosis (*P* < 0.001).
