# Supplementary material for: Effects of socioeconomic status on esophageal adenocarcinoma stage at diagnosis, receipt of treatment, and survival: A population-based cohort study
Source: PLoS One. 2017 Oct 11;12(10):e0186350. doi: 10.1371/journal.pone.0186350 (PMC5636169; doi:10.1371/journal.pone.0186350)
Supplement: S6 Table — (DOCX) [file pone.0186350.s007.docx]

**S6 Table. Odds of EAC stage among people diagnosed with esophageal adenocarcinoma by income quintile, 1993-2012: Multiple imputation method**

| **Variable** | **Cancer stage at EAC diagnosis^*^** | | | | | | | |
| --- | --- | --- | --- | --- | --- | --- | --- | --- |
|  | **Stage II** |  |  | **Stage III** | |  | **Stage IV** | |
|  | **OR (95% CI)** | ***P*-value** |  | **OR (95% CI)** | ***P*-value** |  | **OR (95% CI)** | ***P*-value** |
| Income quintile |  |  |  |  |  |  |  |  |
| Q1 (lowest) | 1.06 (0.66-1.70) | 0.814 |  | 0.81 (0.51-1.28) | 0.368 |  | 1.24 (0.80-1.92) | 0.344 |
| Q2 | 1.13 (0.70-1.82) | 0.616 |  | 0.95 (0.60-1.51) | 0.826 |  | 1.39 (0.89-2.17) | 0.147 |
| Q3 | 1.22 (0.76-1.97) | 0.414 |  | 0.96 (0.60-1.53) | 0.863 |  | 1.22 (0.78-1.91) | 0.384 |
| Q4 | 0.98 (0.62-1.54) | 0.916 |  | 0.77 (0.50-1.20) | 0.246 |  | 0.90 (0.59-1.37) | 0.622 |
| Q5 (highest) | Reference |  |  | Reference |  |  | Reference |  |

Total N = 3,374

^*^Multinomial logistic regression analysis (fully-adjusted model) overall *P*-values: income quintile (*P* = 0.118), age (*P* = 0.011), gender (*P* = 0.578), residence (*P* = 0.154), birth country (*P* = 0.037), Ontario health region (*P* < 0.001), Aggregated Diagnosis Group (ADG) (*P* = 0.048) and year of EAC diagnosis (*P* = 0.014).
