# Supplementary material for: Effects of socioeconomic status on esophageal adenocarcinoma stage at diagnosis, receipt of treatment, and survival: A population-based cohort study
Source: PLoS One. 2017 Oct 11;12(10):e0186350. doi: 10.1371/journal.pone.0186350 (PMC5636169; doi:10.1371/journal.pone.0186350)
Supplement: S7 Table — (DOCX) [file pone.0186350.s008.docx]

**S7 Table. Odds of EAC treatment among people diagnosed with esophageal adenocarcinoma by income quintile, 1993-2012: Multiple imputation method**

| **Variable** | **EAC treatment after diagnosis** | | | | | | | | | | |
| --- | --- | --- | --- | --- | --- | --- | --- | --- | --- | --- | --- |
|  | **Surgery** | |  | **Chemotherapy** | |  | **Radiotherapy** | |  | **Surgery + Chemotherapy** | |
|  | **OR (95% CI)** | ***P*-value** |  | **OR (95% CI)** | ***P*-value** |  | **OR (95% CI)** | ***P*-value** |  | **OR (95% CI)** | ***P*-value** |
| Income quintile |  |  |  |  |  |  |  |  |  |  |  |
| Q1 (lowest) | 0.85 (0.64-1.12) | 0.242 |  | 0.58 (0.41-0.81) | **0.002** |  | 0.60 (0.42-0.86) | **0.005** |  | 0.66 (0.45-0.96) | **0.030** |
| Q2 | 0.82 (0.62-1.09) | 0.170 |  | 0.74 (0.53-1.03) | 0.070 |  | 0.58 (0.41-0.83) | **0.003** |  | 0.60 (0.41-0.88) | **0.009** |
| Q3 | 1.02 (0.78-1.34) | 0.886 |  | 0.82 (0.59-1.15) | 0.256 |  | 0.69 (0.48-1.00) | **0.047** |  | 0.88 (0.61-1.28) | 0.512 |
| Q4 | 1.05 (0.79-1.38) | 0.755 |  | 0.76 (0.54-1.06) | 0.103 |  | 0.60 (0.42-0.87) | **0.008** |  | 0.88 (0.61-1.28) | 0.513 |
| Q5 (highest) | Reference |  |  | Reference |  |  | Reference |  |  | Reference |  |
|  | **Surgery + Radiotherapy** | |  | **Chemotherapy + Radiotherapy** | |  | **Surgery + Chemotherapy + Radiotherapy** | |  |  | |
|  | **OR (95% CI)** | ***P*-value** |  | **OR (95% CI)** | ***P*-value** |  | **OR (95% CI)** | ***P*-value** |  |  |  |
| Income quintile |  |  |  |  |  |  |  |  |  |  |  |
| Q1 (lowest) | 2.44 (0.64-9.32) | 0.191 |  | 0.77 (0.55-1.07) | 0.119 |  | 0.60 (0.41-0.87) | **0.007** |  |  |  |
| Q2 | 2.72 (0.73-10.21) | 0.138 |  | 0.79 (0.57-1.10) | 0.168 |  | 0.69 (0.48-1.00) | **0.048** |  |  |  |
| Q3 | 3.25 (0.88-11.99) | 0.077 |  | 1.03 (0.74-1.44) | 0.850 |  | 0.95 (0.66-1.37) | 0.775 |  |  |  |
| Q4 | 3.91 (1.1-13.94) | **0.035** |  | 0.88 (0.63-1.23) | 0.459 |  | 0.85 (0.59-1.22) | 0.381 |  |  |  |
| Q5 (highest) | Reference |  |  | Reference |  |  | Reference |  |  |  |  |

Total N = 5,382

^*^Multinomial logistic regression analysis (fully-adjusted model) overall *P*-values: income quintile (*P* = 0.103), age (*P* < 0.001), gender (*P* = 0.020), residence (*P* = 0.034), birth country (*P* = 0.052), Ontario health region (*P* < 0.001), Aggregated Diagnosis Group (ADG) (*P* = 0.004), cancer stage at EAC diagnosis (*P* < 0.001) and year of EAC diagnosis (*P* < 0.001).
