# Supplementary material for: Effects of socioeconomic status on esophageal adenocarcinoma stage at diagnosis, receipt of treatment, and survival: A population-based cohort study
Source: PLoS One. 2017 Oct 11;12(10):e0186350. doi: 10.1371/journal.pone.0186350 (PMC5636169; doi:10.1371/journal.pone.0186350)
Supplement: S8 Table — (DOCX) [file pone.0186350.s009.docx]

**S8 Table. Risk of mortality after the diagnosis of esophageal adenocarcinoma, 1993-2012: Cox proportional-hazards regression models: Multiple imputation method**

| **Characteristics** | **Univariate Analysis** | | |  | **Multivariate Analysis** | |
| --- | --- | --- | --- | --- | --- | --- |
|  | **Hazard Ratio (95% CI)** | | ***P*-value** |  | **Hazard Ratio (95% CI)** | ***P*-value** |
| Income quintile |  |  | |  |  |  |
| 1 (lowest) | 1.20 (1.09-1.31) | **<0.001** | |  | 1.17 (1.06-1.28) | **0.001** |
| 2 | 1.15 (1.05-1.26) | **0.003** | |  | 1.11 (1.01-1.22) | **0.031** |
| 3 | 1.11 (1.01-1.22) | **0.033** | |  | 1.10 (1.00-1.21) | 0.052 |
| 4 | 1.04 (0.94-1.14) | 0.478 | |  | 1.05 (0.95-1.15) | 0.362 |
| 5 (highest) | Reference |  | |  | Reference |  |
| Age group (years) |  |  | |  |  |  |
| <50 | Reference |  | |  | Reference |  |
| 50-54 | 0.96 (0.83-1.10) | 0.539 | |  | 0.98 (0.85-1.14) | 0.814 |
| 55-59 | 0.98 (0.86-1.13) | 0.810 | |  | 1.02 (0.89-1.17) | 0.755 |
| 60-64 | 0.93 (0.82-1.06) | 0.304 | |  | 0.97 (0.85-1.11) | 0.673 |
| 65-69 | 1.06 (0.93-1.20) | 0.388 | |  | 1.10 (0.97-1.25) | 0.140 |
| 70-74 | 1.19 (1.05-1.35) | **0.007** | |  | 1.23 (1.08-1.40) | **0.002** |
| 75-79 | 1.32 (1.16-1.50) | **<0.001** | |  | 1.35 (1.18-1.54) | **<0.001** |
| 80-84 | 1.69 (1.48-1.94) | **<0.001** | |  | 1.69 (1.47-1.96) | **<0.001** |
| >85 | 1.95 (1.68-2.27) | **<0.001** | |  | 1.98 (1.69-2.33) | **<0.001** |
| Sex |  |  | |  |  |  |
| Male | Reference |  | |  | Reference |  |
| Female | 1.12 (1.03-1.21) | **0.006** | |  | 1.03 (0.95-1.11) | 0.545 |
| Residence |  |  | |  |  |  |
| Rural | Reference |  | |  | Reference |  |
| Urban | 1.00 (0.93-1.08) | 0.913 | |  | 0.99 (0.91-1.07) | 0.756 |
| Birth country |  |  | |  |  |  |
| Outside of Canada | Reference |  | |  | Reference |  |
| Canada | 0.97 (0.90-1.03) | 0.313 | |  | 0.99 (0.93-1.07) | 0.851 |

S8 Table continued on the following page

**S8 Table. Risk of mortality after the diagnosis of esophageal adenocarcinoma, 1993-2012: Cox proportional-hazards regression models: Multiple imputation method (continued)**

| **Characteristics** | **Univariate Analysis** | | |  | **Multivariate Analysis** | |
| --- | --- | --- | --- | --- | --- | --- |
|  | **Hazard Ratio (95% CI)** | | ***P*-value** |  | **Hazard Ratio (95% CI)** | ***P*-value** |
| Ontario Health Region |  |  | |  |  |  |
| Central | Reference |  | |  | Reference |  |
| Erie St. Clair | 1.09 (0.92-1.30) | 0.317 | |  | 1.07 (0.90-1.27) | 0.436 |
| South West | 1.31 (1.13-1.51) | **<0.001** | |  | 1.23 (1.06-1.44) | **0.007** |
| Waterloo Wellington | 1.12 (0.95-1.32) | 0.181 | |  | 1.14 (0.97-1.35) | 0.118 |
| Hamilton Niagara Haldimand Brant | 1.22 (1.07-1.40) | **0.003** | |  | 1.21 (1.05-1.39) | **0.007** |
| Central West | 0.96 (0.79-1.17) | 0.710 | |  | 1.01 (0.83-1.24) | 0.911 |
| Mississauga | 1.15 (0.96-1.38) | 0.124 | |  | 1.13 (0.95-1.36) | 0.169 |
| Toronto Central | 1.14 (0.97-1.34) | 0.101 | |  | 1.12 (0.95-1.31) | 0.178 |
| Central East | 1.19 (1.03-1.37) | **0.018** | |  | 1.16 (1.00-1.34) | 0.048 |
| South East | 1.12 (0.96-1.31) | 0.140 | |  | 1.00 (0.86-1.18) | 0.974 |
| Champlain | 1.07 (0.93-1.24) | 0.336 | |  | 1.01 (0.87-1.16) | 0.939 |
| North Simcoe | 0.99 (0.83-1.18) | 0.923 | |  | 0.96 (0.80-1.15) | 0.657 |
| North East | 1.15 (0.98-1.35) | 0.080 | |  | 1.22 (1.04-1.44) | **0.017** |
| North West | 1.03 (0.83-1.27) | 0.809 | |  | 1.07 (0.86-1.33) | 0.530 |
| ADG |  |  | |  |  |  |
| 0 | Reference |  | |  | Reference |  |
| 1-3 | 0.99 (0.68-1.42) | 0.935 | |  | 1.12 (0.78-1.62) | 0.547 |
| 4-7 | 0.91 (0.64-1.29) | 0.587 | |  | 0.98 (0.69-1.39) | 0.903 |
| 8-10 | 0.86 (0.61-1.22) | 0.401 | |  | 0.91 (0.64-1.29) | 0.606 |
| 11+ | 0.89 (0.63-1.26) | 0.506 | |  | 0.89 (0.63-1.26) | 0.517 |

S8 Table continued on the following page

**S8 Table. Risk of mortality after the diagnosis of esophageal adenocarcinoma, 1993-2012: Cox proportional-hazards regression models: Multiple imputation method (continued)**

| **Characteristics** | **Univariate Analysis** | | |  | **Multivariate Analysis** | |
| --- | --- | --- | --- | --- | --- | --- |
|  | **Hazard Ratio (95% CI)** | | ***P*-value** |  | **Hazard Ratio (95% CI)** | ***P*-value** |
| Stage at EAC diagnosis^*^ |  |  | |  |  |  |
| Stage 0-I | Reference |  | |  | Reference |  |
| Stage II | 1.09 (0.96-1.24) | 0.208 | |  | 1.01 (0.88-1.15) | 0.909 |
| Stage III | 1.16 (1.02-1.32) | **0.020** | |  | 1.11 (0.98-1.26) | 0.110 |
| Stage IV | 1.66 (1.47-1.87) | **<0.001** | |  | 1.53 (1.35-1.72) | **<0.001** |
| EAC treatment^*^ |  |  | |  |  |  |
| Surgery (yes vs. no) | 0.56 (0.52-0.61) | **<0.001** | |  | 0.62 (0.56-0.67) | **<0.001** |
| Chemotherapy (yes vs. no) | 1.20 (1.11-1.30) | **<0.001** | |  | 1.21 (1.09-1.34) | **<0.001** |
| Radiotherapy (yes vs. no) | 1.19 (1.11-1.29) | **<0.001** | |  | 1.36 (1.20-1.53) | **<0.001** |
| Surgery + chemotherapy (yes vs. no) | 0.81 (0.73-0.90) | **<0.001** | |  | 1.20 (1.06-1.35) | **0.003** |
| Surgery + radiotherapy (yes vs. no) | 0.94 (0.63-1.40) | 0.751 | |  | 1.00 (0.66-1.52) | 0.989 |
| Chemotherapy + radiotherapy (yes vs. no) | 0.99 (0.92-1.08) | 0.866 | |  | 0.97 (0.86-1.10) | 0.675 |
| Surgery + chemotherapy + radiotherapy (yes vs. no) | 0.65 (0.57-0.73) | <0.001 | |  | 0.74 (0.64-0.85) | <.0001 |
| Year of EAC diagnosis |  |  | |  |  |  |
| 1993-1997 | 1.22 (1.11-1.33) | **<0.001** | |  | 1.37 (1.23-1.52) | **<0.001** |
| 1998-2002 | 1.14 (1.05-1.24) | **0.002** | |  | 1.25 (1.13-1.38) | **<0.001** |
| 2003-2007 | 1.13 (1.05-1.22) | **0.002** | |  | 1.17 (1.08-1.27) | **<0.001** |
| 2008-2012 | Reference |  | |  | Reference |  |

Total N = 5,380. ^*^Variable modeled as time-dependent covariate. ADG, Aggregated Diagnosis Group; EAC, esophageal adenocarcinoma. Univariate (unadjusted model,) analysis overall *P*-values: income quintile (*P* < 0.001), age (*P* < 0.001), Ontario health region (*P* = 0.006), ADG (*P* = 0.372), cancer stage at EAC diagnosis (*P* < 0.001) and year of EAC diagnosis (*P* < 0.001). Multivariate (fully-adjusted model) analysis overall *P*-values: income quintile (*P* = 0.017), age (*P* < 0.001), Ontario health region (*P* = 0.004), ADG (*P* = 0.011), cancer stage at EAC diagnosis (*P* < 0.001) and year of EAC diagnosis (*P* < 0.001).
