# Supplementary material for: Effects of socioeconomic status on esophageal adenocarcinoma stage at diagnosis, receipt of treatment, and survival: A population-based cohort study
Source: PLoS One. 2017 Oct 11;12(10):e0186350. doi: 10.1371/journal.pone.0186350 (PMC5636169; doi:10.1371/journal.pone.0186350)
Supplement: S9 Table — (DOCX) [file pone.0186350.s010.docx]

**S9 Table. Risk of mortality after the diagnosis of esophageal adenocarcinoma, 1993-2012: Cox proportional-hazards regression models: Multiple imputation, excluding advanced-stage IV**

| **Characteristics** | **Univariate Analysis** | | |  | **Multivariate Analysis** | |
| --- | --- | --- | --- | --- | --- | --- |
|  | **Hazard Ratio (95% CI)** | | ***P*-value** |  | **Hazard Ratio (95% CI)** | ***P*-value** |
| Income quintile |  |  | |  |  |  |
| 1 (lowest) | 1.24 (1.09-1.42) | **0.001** | |  | 1.26 (1.10-1.44) | **0.001** |
| 2 | 1.20 (1.05-1.37) | **0.007** | |  | 1.22 (1.06-1.39) | **0.004** |
| 3 | 1.19 (1.05-1.36) | **0.008** | |  | 1.19 (1.05-1.36) | **0.009** |
| 4 | 1.10 (0.96-1.25) | 0.171 | |  | 1.15 (1.01-1.31) | **0.043** |
| 5 (highest) | Reference |  | |  | Reference |  |
| Age group (years) |  |  | |  |  |  |
| <50 | Reference |  | |  | Reference |  |
| 50-54 | 0.96 (0.77-1.19) | 0.696 | |  | 0.90 (0.72-1.12) | 0.333 |
| 55-59 | 1.00 (0.81-1.23) | 0.994 | |  | 1.00 (0.81-1.23) | 0.961 |
| 60-64 | 0.89 (0.73-1.10) | 0.279 | |  | 0.91 (0.74-1.12) | 0.362 |
| 65-69 | 1.08 (0.89-1.32) | 0.424 | |  | 1.06 (0.87-1.29) | 0.572 |
| 70-74 | 1.28 (1.05-1.55) | **0.013** | |  | 1.23 (1.01-1.50) | **0.045** |
| 75-79 | 1.53 (1.26-1.86) | **<0.001** | |  | 1.52 (1.24-1.87) | **<0.001** |
| 80-84 | 1.77 (1.44-2.18) | **<0.001** | |  | 1.69 (1.36-2.11) | **<0.001** |
| >85 | 2.44 (1.97-3.01) | **<0.001** | |  | 2.34 (1.86-2.94) | **<0.001** |
| Sex |  |  | |  |  |  |
| Male | Reference |  | |  | Reference |  |
| Female | 1.20 (1.08-1.33) | **0.001** | |  | 1.01 (0.90-1.12) | 0.896 |
| Residence |  |  | |  |  |  |
| Rural | Reference |  | |  | Reference |  |
| Urban | 0.96 (0.87-1.07) | 0.469 | |  | 0.95 (0.84-1.06) | 0.323 |
| Birth country |  |  | |  |  |  |
| Outside of Canada | Reference |  | |  | Reference |  |
| Canada | 0.87 (0.80-0.96) | **0.004** | |  | 0.96 (0.87-1.06) | 0.416 |

S9 Table continued on the following page

**S9 Table. Risk of mortality after the diagnosis of esophageal adenocarcinoma, 1993-2012: Cox proportional-hazards regression models: Multiple imputation, excluding advanced-stage IV (continued)**

| **Characteristics** | **Univariate Analysis** | | |  | **Multivariate Analysis** | |
| --- | --- | --- | --- | --- | --- | --- |
|  | **Hazard Ratio (95% CI)** | | ***P*-value** |  | **Hazard Ratio (95% CI)** | ***P*-value** |
| Ontario Health Region |  |  | |  |  |  |
| Central | Reference |  | |  | Reference |  |
| Erie St. Clair | 1.04 (0.82-1.33) | 0.730 | |  | 1.14 (0.89-1.46) | 0.297 |
| South West | 1.20 (0.97-1.49) | 0.092 | |  | 1.17 (0.93-1.46) | 0.180 |
| Waterloo Wellington | 1.06 (0.85-1.32) | 0.636 | |  | 1.10 (0.88-1.39) | 0.397 |
| Hamilton Niagara Haldimand Brant | 1.08 (0.89-1.31) | 0.429 | |  | 1.16 (0.95-1.41) | 0.139 |
| Central West | 1.01 (0.78-1.31) | 0.936 | |  | 1.08 (0.83-1.40) | 0.586 |
| Mississauga | 1.10 (0.86-1.41) | 0.448 | |  | 1.11 (0.87-1.43) | 0.399 |
| Toronto Central | 1.17 (0.95-1.44) | 0.145 | |  | 1.15 (0.93-1.42) | 0.199 |
| Central East | 1.13 (0.93-1.37) | 0.205 | |  | 1.12 (0.92-1.36) | 0.258 |
| South East | 1.03 (0.84-1.27) | 0.770 | |  | 0.94 (0.75-1.17) | 0.552 |
| Champlain | 0.91 (0.74-1.12) | 0.367 | |  | 0.89 (0.72-1.10) | 0.289 |
| North Simcoe | 1.06 (0.84-1.34) | 0.605 | |  | 1.04 (0.82-1.31) | 0.776 |
| North East | 1.10 (0.89-1.36) | 0.368 | |  | 1.20 (0.96-1.50) | 0.108 |
| North West | 0.82 (0.60-1.11) | 0.191 | |  | 0.95 (0.69-1.29) | 0.720 |
| ADG |  |  | |  |  |  |
| 0 | Reference |  | |  | Reference |  |
| 1-3 | 0.95 (0.48-1.89) | 0.887 | |  | 0.94 (0.47-1.87) | 0.857 |
| 4-7 | 0.96 (0.50-1.86) | 0.901 | |  | 0.88 (0.45-1.73) | 0.717 |
| 8-10 | 0.91 (0.47-1.76) | 0.779 | |  | 0.84 (0.43-1.63) | 0.596 |
| 11+ | 0.98 (0.51-1.89) | 0.953 | |  | 0.82 (0.42-1.60) | 0.558 |

S9 Table continued on the following page

**S9 Table. Risk of mortality after the diagnosis of esophageal adenocarcinoma, 1993-2012: Cox proportional-hazards regression models: Multiple imputation, excluding advanced-stage IV (continued)**

| **Characteristics** | **Univariate Analysis** | | |  | **Multivariate Analysis** | |
| --- | --- | --- | --- | --- | --- | --- |
|  | **Hazard Ratio (95% CI)** | | ***P*-value** |  | **Hazard Ratio (95% CI)** | ***P*-value** |
| Stage at EAC diagnosis^*^ |  |  | |  |  |  |
| Stage 0-I | Reference |  | |  | Reference |  |
| Stage II | 1.09 (0.96-1.24) | 0.184 | |  | 1.04 (0.91-1.19) | 0.548 |
| Stage III | 1.17 (1.03-1.33) | **0.015** | |  | 1.12 (0.98-1.27) | 0.087 |
| EAC treatment^*^ |  |  | |  |  |  |
| Surgery (yes vs. no) | 0.61 (0.55-0.68) | **<0.001** | |  | 0.65 (0.58-0.74) | **<0.001** |
| Chemotherapy (yes vs. no) | 1.10 (0.98-1.23) | 0.122 | |  | 1.18 (1.01-1.37) | **0.039** |
| Radiotherapy (yes vs. no) | 0.98 (0.88-1.10) | 0.764 | |  | 1.17 (0.98-1.40) | 0.087 |
| Surgery + chemotherapy (yes vs. no) | 0.80 (0.70-0.91) | **0.001** | |  | 1.27 (1.08-1.50) | **0.004** |
| Surgery + radiotherapy (yes vs. no) | 1.00 (0.64-1.58) | 0.987 | |  | 1.20 (0.74-1.95) | 0.460 |
| Chemotherapy + radiotherapy (yes vs. no) | 0.85 (0.76-0.96) | **0.007** | |  | 1.06 (0.88-1.29) | 0.521 |
| Surgery + chemotherapy + radiotherapy (yes vs. no) | 0.67 (0.58-0.77) | **<0.001** | |  | 0.85 (0.71-1.01) | 0.066 |
| Year of EAC diagnosis |  |  | |  |  |  |
| 1993-1997 | 1.69 (1.49-1.91) | **<0.001** | |  | 1.91 (1.64-2.23) | **<0.001** |
| 1998-2002 | 1.59 (1.41-1.79) | **<0.001** | |  | 1.77 (1.54-2.03) | **<0.001** |
| 2003-2007 | 1.25 (1.12-1.40) | **<0.001** | |  | 1.33 (1.18-1.49) | **<0.001** |
| 2008-2012 | Reference |  | |  | Reference |  |

Total N = 2,847. ^*^Variable modeled as time-dependent covariate. ADG, Aggregated Diagnosis Group; EAC, esophageal adenocarcinoma. Univariate (unadjusted model,) analysis overall *P*-values: income quintile (*P* = 0.009), age (*P* < 0.001), Ontario health region (*P* = 0.281), ADG (*P* = 0.719), cancer stage at EAC diagnosis (*P* = 0.038) and year of EAC diagnosis (*P* < 0.001). Multivariate (fully-adjusted model) analysis overall *P*-values: income quintile (*P* = 0.010), age (*P* < 0.001), Ontario health region (*P* = 0.139), ADG (*P* = 0.569), cancer stage at EAC diagnosis (*P* = 0.136) and year of EAC diagnosis (*P* < 0.001).
